# Synthesis and Evaluation of Indatraline-Based Inhibitors for Trypanothione Reductase

**DOI:** 10.1002/cmdc.201000442

**Published:** 2010-12-15

**Authors:** Jeffrey G A Walton, Deuan C Jones, Paula Kiuru, Alastair J Durie, Nicholas J Westwood, Alan H Fairlamb

**Affiliations:** [a] School of Chemistry and Biomedical Sciences Research Complex, University of St AndrewsNorth Haugh, St Andrews, Fife, KY16 9ST (UK); [b] Division of Biological Chemistry and Drug Discovery, University of DundeeDundee, DD1 5EH (UK)

**Keywords:** antiprotozoal agents, drug discovery, indatraline, Nazarov reaction, trypanothione reductase

## Abstract

The search for novel compounds of relevance to the treatment of diseases caused by trypanosomatid protozoan parasites continues. Screening of a large library of known bioactive compounds has led to several drug-like starting points for further optimisation. In this study, novel analogues of the monoamine uptake inhibitor indatraline were prepared and assessed both as inhibitors of trypanothione reductase (TryR) and against the parasite *Trypanosoma brucei*. Although it proved difficult to significantly increase the potency of the original compound as an inhibitor of TryR, some insight into the preferred substituent on the amine group and in the two aromatic rings of the parent indatraline was deduced. In addition, detailed mode of action studies indicated that two of the inhibitors exhibit a mixed mode of inhibition.

## Introduction

Trypanosomatid protozoan parasites cause a variety of important diseases, including human African sleeping sickness, Chagas disease, and leishmaniasis. Sleeping sickness is caused by *Trypanosoma brucei* ssp., and is endemic in certain regions of sub-Saharan Africa, covering about 20countries with an estimated 70–80 000people infected.^1^ Chagas disease is present in 19countries on the American continent and is responsible for disease in around 8–11million people.^1^ The causative agent of this disease is the parasite *Trypanosoma cruzi*. Leishmaniasis is caused by members of the genus *Leishmania* and is found in 88countries, threatening 350million people.^1^ In combination, these diseases contribute to approximately 95 000deaths annually, but considering their socioeconomic importance, the current scale of new drug discovery and vaccine development efforts is inadequate. In addition, the available therapies are often toxic, marginally effective, administered by injection, and expensive. In particular, there is an urgent need for new CNS-active drugs to treat late-stage sleeping sickness to replace the current therapies that are losing efficacy due to parasite resistance.^1^

**Figure 1:**
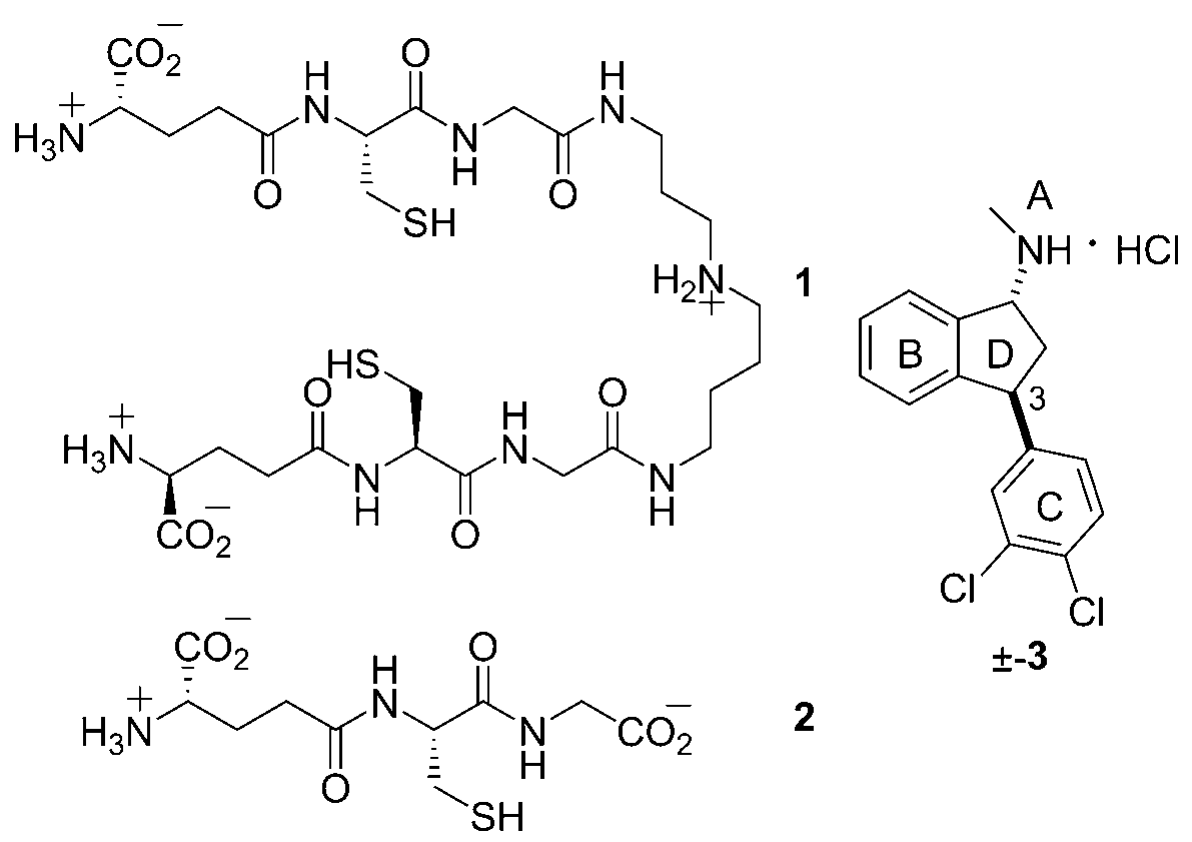
Structures of trypanothione (**1**), glutathione (**2**), and indatraline (**3**).

The trypanosomatids use a polyamine–glutathione adduct, trypanothione (**1**, Figure 1), as a key component of their defence system. Compound **1** is prepared through a unique biosynthetic pathway in which glutathione (**2**) is conjugated to spermidine.^2^ In humans, glutathione and glutathione reductase (GR) are used to maintain the intracellular redox balance, whereas the analogous chemistry in the parasite is carried out by trypanothione reductase (TryR), which reduces trypanothione disulfide (T[S]_2_) to **1**. Previous genetic knockout studies have illustrated the essential role of TryR in parasite viability,^3^ validating it as a target for drug development in all three diseases. Importantly, comparison of TryR and human GR crystal structures reveal significant differences between their active sites,^4^ suggesting that these differences may be exploited to gain selectivity for TryR over GR.

As part of a concerted campaign to discover new treatments for trypanosomatid-based diseases, we undertook a high-throughput screen for inhibitors of TryR. The Sigma-LOPAC^1280^ collection, a library of compounds with known pharmacological activity, was screened against TryR.^5^ The thinking behind screening a library of known drugs is encapsulated in Sir James Black’s famous quote: “The most fruitful basis for the discovery of a new drug is to start with an old drug”.^6^ It was planned that hits derived from small molecules that already have desirable drug-like properties could be modified to tune their selectivity away from their original protein targets and towards TryR without too much disruption of the desirable drug-like properties.

As reported previously,^5^ assessment of initial screening hits against human GR and *T. brucei* cells together with in silico analysis of chemical properties revealed three new classes of TryR inhibitors that merited further development. Investigation of one of these classes, based on 1-[1-(2-benzo[*b*]thienyl)cyclohexyl)]piperidine (BTCP), was reported previously.^7^ Herein we describe a related investigation of a further class of compounds based on indatraline (**3**), a nonselective monoamine reuptake inhibitor.^8^ The molecule contains an indanamine core structure with a second aromatic unit at the 3-position (Figure 1). This CNS-active molecule was considered suitable for extensive modification and was of sufficiently low molecular weight to allow adherence to Lipinski’s rules, even following considerable alteration.^9^ We identified four possible sites (A–D, Figure 1) for modification of the core structure. Details of the parallel synthetic routes that were developed along with careful analysis of the activity of the analogues generated against TryR in vitro and *T. brucei* in culture are reported. Whilst it proved difficult in this chemical series to improve potency against the desired target, a new important insight into the mode of inhibition of TryR by these analogues was discovered, progressing our thinking on how to inhibit effectively this important enzyme.

## Results and Discussion

### Synthesis of indatraline analogues

**Scheme 1:**
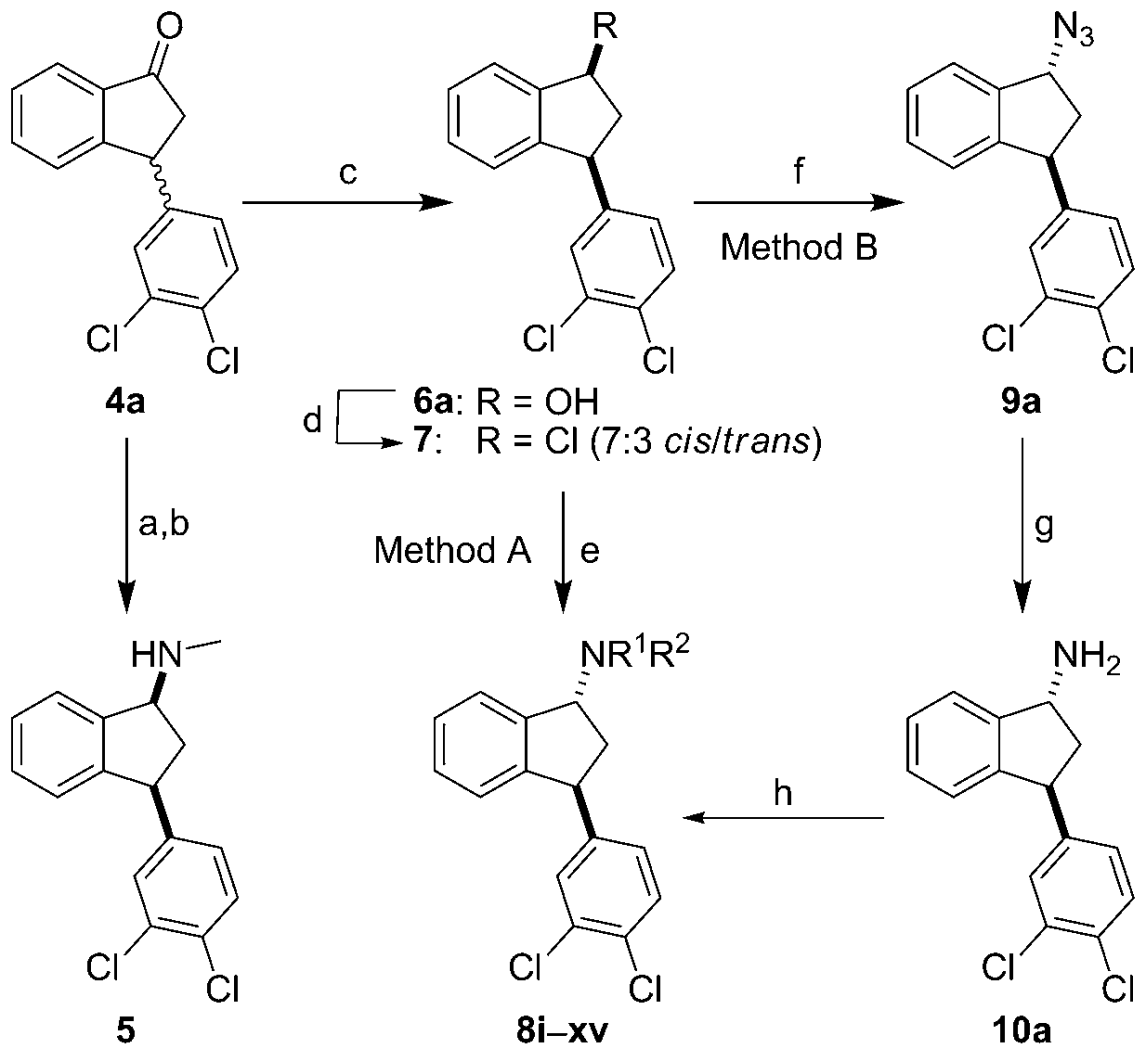
Reagents and conditions: a) MeNH_2_, TiCl_4_, PhMe, −10 °C, 1h; b) NaBH_4_, MeOH, RT, 3h (62 %); c) NaBH_4_, MeOH, RT, 2h (77 %); d) SOCl_2_, Tol., RT, 3h; e) NHR^1^R^2^, THF, 90 °C, 4h; f) (PhO)_2_P(O)N_3_, DBU, THF, RT, o/n (93 %); g) PS–PPh_3_, H_2_O, THF, RT, 16h (quant); h) R^1^CHO, NaBH(OAc)_3_ or CH_3_COCl or TsCl, THF, RT, o/n. R^1^ and R^2^ are defined in Table 1 and Supporting Information.^11^

Initial studies focused on the amino substituent in **3** (site A, Figure 1) starting from the common intermediate 3-phenylindanone (**4a**, Scheme 1). Compound **4a** was prepared according to published methods.^10^ Treatment of **4a** with methylamine in the presence of titanium tetrachloride followed by reduction of the resulting imine with sodium borohydride afforded indanamine **5** as the *cis* isomer, as reported by Bøgesø et al.8a Access to the *trans*-indanamines was achieved as follows:8a Reduction of indanone **4a** with sodium borohydride gave 3-phenylindan-1-ol (**6a**) in high yield and high *cis* selectivity (97:3). A single recrystallisation was required to afford the pure *cis* isomer. Reaction of **6a** with thionyl chloride resulted in an isomeric mixture of *cis*- and *trans*-1-chloro-3-phenylindanes (**7**), with a *cis*/*trans* ratio of 7:3. Crude **7** was then reacted with a series of primary and secondary alkylamines to produce the corresponding 3-phenylindan-1-amines with, as expected, a reversal of the *cis*/*trans* ratio (3:7). The pure *trans* isomers **8i**–**vi** were isolated following purification by semi-preparative HPLC, and the stereochemistry was assigned by comparison with published work.8a

Having prepared analogues **5** and **8i**–**vi**, we decided to evaluate the routes used for conversion into a parallel synthesis protocol. This was viewed as challenging due to the required separation of the isomeric mixtures of **8** on a small scale. We therefore decided to adapt the original route by incorporating a modified Mitsunobu protocol to convert indanol **6a** to the azide **9a** (Scheme 1).^12^ This reaction occurred with complete inversion of the C1stereochemistry in **6a** and generated exclusively the *trans* isomer of **9a**, removing the requirement to separate isomeric mixtures later in the route. A Staudinger reduction of azide **9a** using polymer-supported triphenylphosphine generated the indanamine **10a** in high yield and purity following filtration to remove the reagent. Reductive amination of **10a** with a range of aldehydes in the presence of sodium triacetoxyborohydride afforded the required *trans*-indanamines **8vii**–**xiii**. Initially, purification of these compounds was necessary, as over-alkylation of **10a** was observed. However when an excess of **10a** (1.2equivalents) was used in this reaction, none of the dialkylated product was formed, and remaining **10a** was removed using a polymer-supported benzaldehyde scavenger resin. With this protocol it was possible to prepare pure *trans*-indanamines **8vii**–**xiii** in moderate to high yields. Indanamine **10a** was also treated with acetyl and tosyl chloride to afford **8xiv** and **8xv**, respectively. The stereochemistry of **8** and **9** prepared by this route was assigned based on literature precedent from Rice and co-workers.^12^

**Scheme 2:**
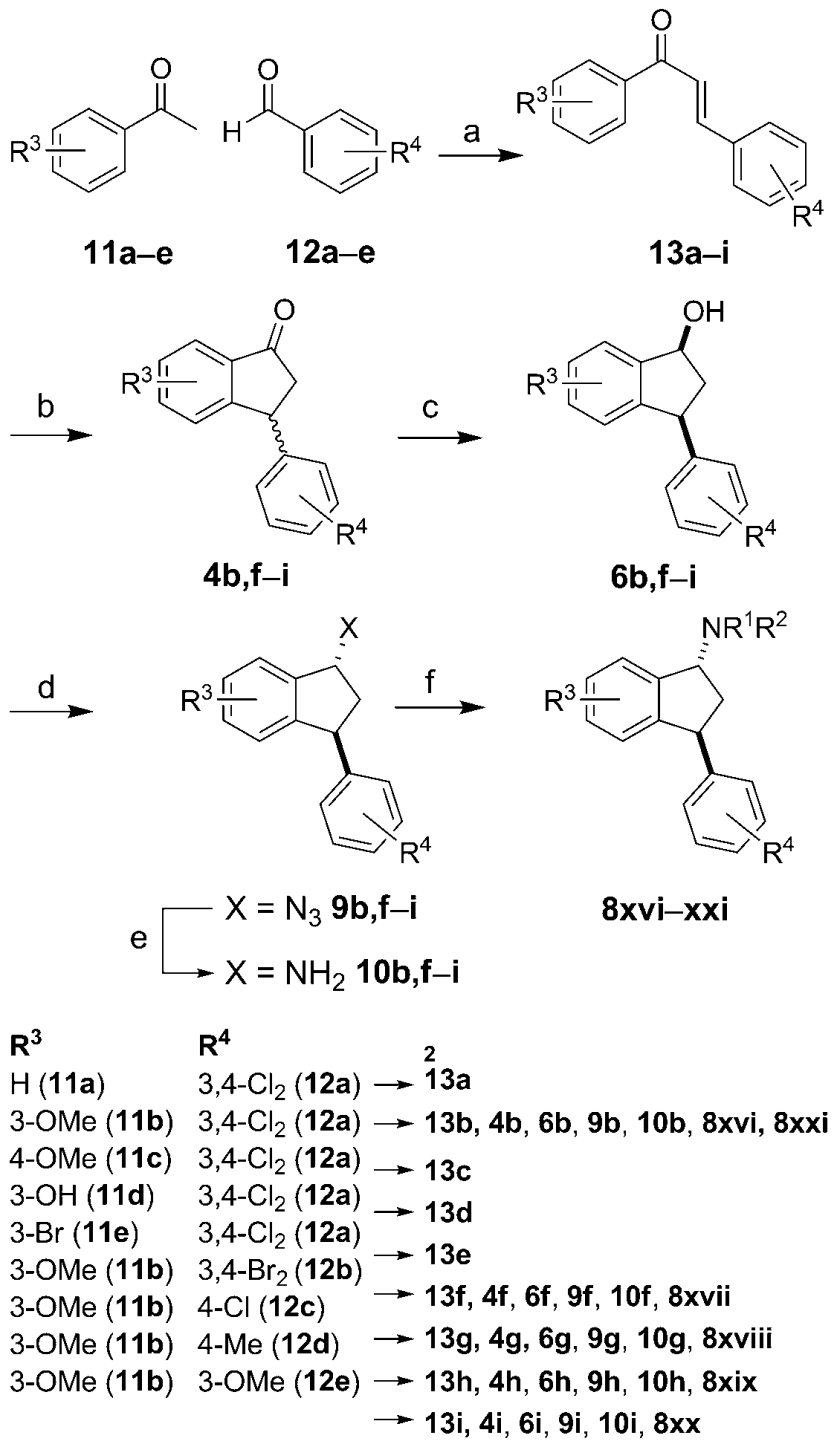
Reagents and conditions: a) R^4^CHO, KOH, EtOH, 0 °C, 2h; b) TFA, 120 °C, microwave, 10min; c) NaBH_4_, MeOH, RT, 2h; d) (PhO)_2_P(O)N_3_, DBU, THF, RT, o/n; e) PS–PPh_3_, H_2_O, THF, RT, 16h; f) R^1^CHO, NaBH(OAc)_3_, THF, RT, o/n. R^1^–R^4^ are defined in Table 1 and Supporting Information.^11^

Having developed a robust route to amino-substituted analogues, we decided to investigate modification of the two aromatic rings present in **3**. Rice and colleagues reported a protocol to access the indanone core via an aldol condensation of 3-methoxyacetophenone (**11b**, Scheme 2) with 3,4-dichlorobenzaldehyde (**12a**) to generate chalcone (**13b**).^12^, ^13^ In their hands, a subsequent Nazarov cyclisation in the presence of trifluoroacetic acid afforded 6-methoxyindanone (**4b**) in high yield. This route was particularly appealing, as it allowed rapid access to the indanone core in two steps. Furthermore, the product from the aldol reaction crystallised out of the reaction solution and required no further purification. These factors made this approach potentially adaptable to a high-throughput synthesis format. To investigate this further, a series of chalcones (compounds **13a**–**e**) were prepared from the aldol reaction between acetophenones **11a**–**e** and 3,4-dichlorobenzaldehyde (**12a**). The chalcones **13a**–**e** were then submitted to cyclisation reactions using trifluoroacetic acid. The Nazarov reaction only occurred when a 3-methoxy substituent was present in the chalcone (for substrate **13b**). In this case, it was possible to decrease the reaction time considerably by carrying out the reactions in a microwave reactor as opposed to conventional heating (10min versus 4h, respectively). Having determined the requirements for a successful Nazarov reaction, a collection of indanones (**4b**,**f**–**i**) was prepared by using 3-methoxyacetophenone (**11b**) and a variety of benzaldehydes **12b**–**e**. The electronic nature of the substituent on the benzaldehydes did not influence the outcome of the Nazarov cyclisation, and all the chalcones afforded the desired indanones **4f**–**i**. These were then converted into the indanamines **8xvi**–**xxi** via method B (Scheme 1).

### Biological analysis against TryR, GR, and *T. brucei*

The analogues prepared according to the methods described above were tested against *T. cruzi* TryR and in an assay for *T. brucei* cell proliferation, as described below in the Experimental Section.

The biological data for a selected group of these compounds are summarised in Table 1. The *cis* isomer **5** (Table 1, Entry 1) was less active against TryR than indatraline **3**, Entry 2). This resulted in the synthetic efforts being focused on the generation of *trans* analogues. As the size and lipophilicity of the amino substituent was increased (Entries 1and 3–6), there appeared to be a relatively flat SAR (see also analogues **S8xxii**–**S8xxvii** in the Supporting Information^11^). This suggests that this region of the molecule contributes little to the biological activity and is probably incorporated in a large open region of the active site. The slightly lower activity of the *N*-*t*Bu analogue **8iii** may, however, indicate some steric constraint close to the nitrogen atom. In addition, the piperidine (**8v**, Entry 7) and morpholine (**8vi**, Entry 8) analogues were prepared. Whilst the piperidine analogue **8v** retained activity, the insertion of an additional oxygen atom, in the case of the morpholine analogue **8vi**, caused a significant decrease in activity (see also analogue **S8xxx** in the Supporting Information^11^). By replacing the methyl substituent of indatraline with a benzyl group (**8vii**, Entry 9), the activity was retained. This provided the opportunity to synthesise a Topliss series of analogues.^14^ Therefore the 4-chloro (**8viii**, Entry 10), 3,4-dichloro (**8ix**, Entry 11), 4-methyl (**8x**, Entry 12), and 4-methoxy (**8xi**, Entry 13) analogues were prepared. The incorporation of electron-donating substituents led to the highest activity, whereas activity diminished when electron-withdrawing groups were introduced. This result led to the preparation of the remaining two compounds in the electron-rich Topliss series: 4-dimethylamino (**8xii**, Entry 14) and 4-amino (**8xiii**, Entry 15). These results further highlight the benefits associated with the inclusion of an electron-donating substituent, although no further improvement in activity was observed relative to the 4-methoxy analogue **8xi**. For data on additional substituted benzyl analogues, see table S1, analogues **S8xxxi**–**S8xxxv** (Supporting Information). The acetyl (**8xiv**, Entry 16) and tosyl (**8xv**, Entry 17) analogues were inactive against TryR (see also analogue **S8xxxvi** in the Supporting Information^11^).

**Table 1:** Biological data for selected indatraline analogues.

Entry	Compound	R^1^	R^2^	R^3^	R^4^	TryR IC_50_ [μm][a]	*T. brucei* EC_50_ [μm]
	Pentamidine[b]						0.0037±0.0001
1	**3**	Me	H	H	3,4-Cl_2_	8.84±0.24	1.06±0.05
2	**5**[c]	Me	H	H	3,4-Cl_2_	13.5±0.7	1.50±0.23
3	**8i**[b]	Et	H	H	3,4-Cl_2_	7.19±0.40	2.12±0.06
4	**8ii**	Isoamyl	H	H	3,4-Cl_2_	4.05±0.39	1.08±0.07
5	**8iii**[b]	*t*Bu	H	H	3,4-Cl_2_	15.3±1.4	0.48±0.04
6	**8iv**	Octyl	H	H	3,4-Cl_2_	7.93±0.78	0.66±0.04
7	**8v**[b]	–CH_2_(CH_2_)_3_CH_2_–		H	3,4-Cl_2_	5.47±0.32	1.31±0.11
8	**8vi**	–(CH_2_)_2_O(CH_2_)_2_–		H	3,4-Cl_2_	173±56	6.62±0.77
9	**8vii**[b]	Benzyl	H	H	3,4-Cl_2_	8.04±0.91	3.53±0.35
10	**8viii**	4-Cl-Bn	H	H	3,4-Cl_2_	17.6±3.3	3.22±0.26
11	**8ix**	3,4-Cl_2_-Bn	H	H	3,4-Cl_2_	45.9±4.2	8.87±0.60
12	**8x**	4-Me-Bn	H	H	3,4-Cl_2_	13.0±1.6	1.29±0.04
13	**8xi**	4-OMe-Bn	H	H	3,4-Cl_2_	4.14±0.41	2.36±0.22
14	**8xii**	4-Me_2_N-Bn	H	H	3,4-Cl_2_	7.38±1.21	1.98±0.23
15	**8xiii**	4-NH_2_-Bn	H	H	3,4-Cl_2_	4.94±0.43	2.68±1.04
16	**8xiv**	Acetyl	H	H	3,4-Cl_2_	>200	3.64±0.28
17	**8xv**	Tosyl	H	H	3,4-Cl_2_	>200	4.33±0.33
18	**8xvi**	Isoamyl	H	6-OMe	3,4-Cl_2_	2.23±0.66	1.26±0.13
19	**8xvii**	Isoamyl	H	6-OMe	3,4-Br_2_	3.15±0.23	2.47±0.25
20	**8xviii**	Isoamyl	H	6-OMe	4-Cl	15.8±1.6	2.51±0.33
21	**8xix**	Isoamyl	H	6-OMe	4-Me	32.9±4.3	1.49±0.08
22	**8xx**	Isoamyl	H	6-OMe	3-OMe	125±17	6.99±1.55
23	**8xxi**	Benzyl	H	6-OMe	3,4-Cl_2_	3.07±0.45	2.30±0.22

[a] Inhibition of trypanothione reductase (TryR).

[b] Pentamidine was included as a reference drug in biological testing.16a

[c] See Ref. 11.

We decided to use the isoamyl group (see **8ii**, Entry 4) as the substituent for site A, whilst an exploration of the B and C rings was carried out. The inclusion of a 6-methoxy substituent in the B ring was investigated, as this was a synthetic requirement for the Nazarov reaction. Analogue **8xvi** (Entry 18) was prepared as a direct comparison with **8ii** (Entry 4). The increased activity of **8xvi** relative to **8ii** demonstrated that the presence of an electron-donating substituent in the B ring is favourable. Therefore a collection of analogues was synthesised containing the 6-methoxy substituent but with variations in substituents in the C ring. The 3,4-dibromo (**8xvii**, Entry 19), 4-chloro (**8xviii**, Entry 20), 4-methyl (**8xix**, Entry 21), and 3-methoxy (**8xx**, entry 22) substituted analogues were prepared and tested. A clear preference for electron-withdrawing substituents in the C ring was observed (see also **8xxi** and additional analogues in table S1, Supporting Information).

All the analogues were also tested against *T. brucei* cultured in vitro. These data predominantly followed a trend similar to that of the IC_50_ data, with decreases in the IC_50_ values being mirrored by corresponding decreases in the EC_50_ values. However, there were a few notable exceptions. Compounds **8iii** (EC_50_=0.48 μm, IC_50_=15.29 μm) and **8iv** (EC_50_=0.66 μm, IC_50_=7.93 μm) showed much improved EC_50_ values relative to their corresponding IC_50_ values. Furthermore, although **8xiv** (EC_50_=3.64 μm, IC_50_>200 μm) and **8xv** (EC_50_=4.33 μm, IC_50_>200 μm) were inactive against TryR, they showed reasonable activity against *T. brucei*. These results could suggest that these analogues have additional off-target effects, or are selectively concentrated/metabolically activated, or a combination of these. In addition, all compounds were tested for inhibition of human GR as described in the Experimental Section below. Compounds were tested in duplicate at a single final concentration of 25 μm. All compounds exhibited <12 % inhibition.

**Figure 2:**
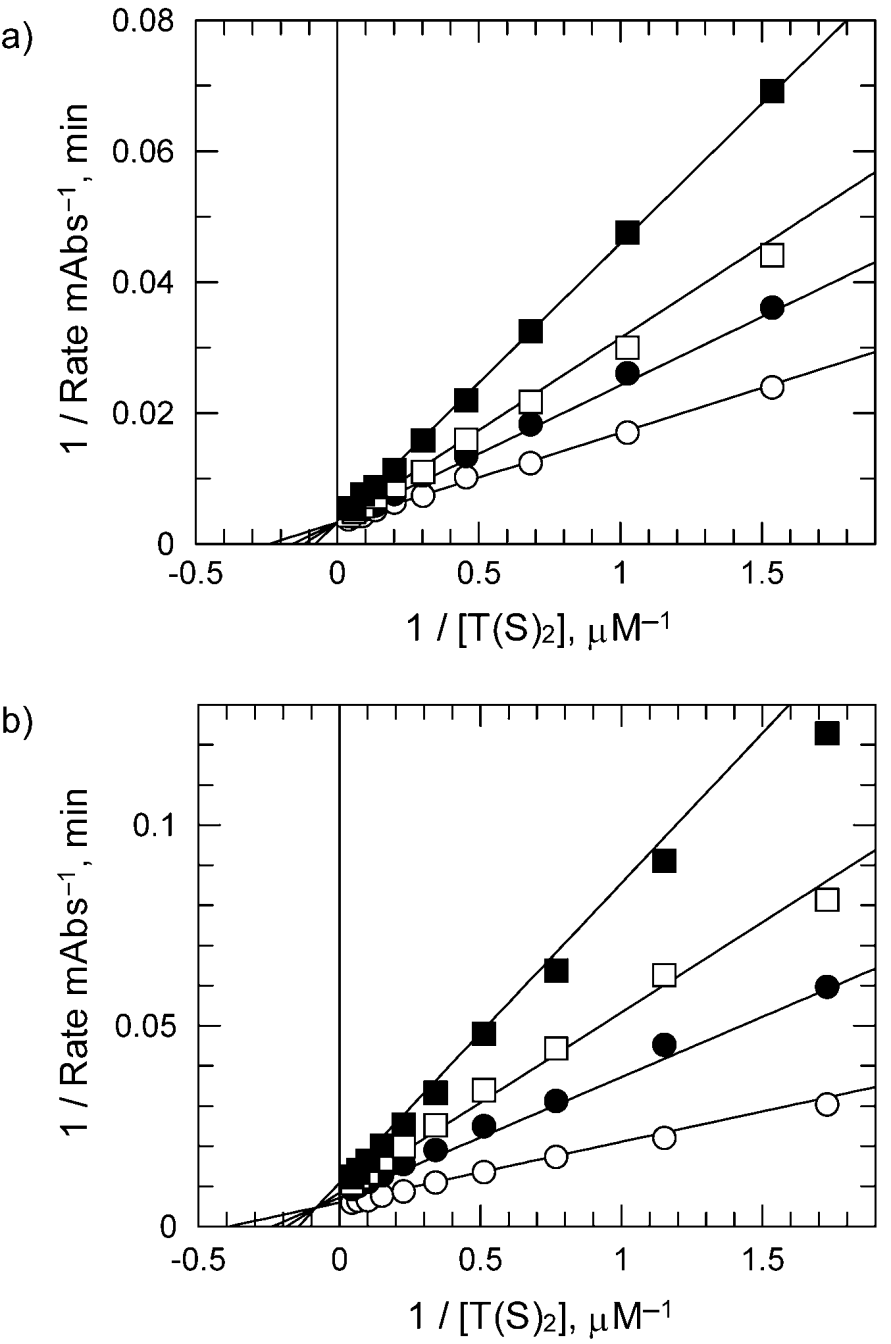
Mode of inhibition by indatraline and compound **8ii**. a) Indatraline: linear competitive inhibition with respect to trypanothione disulfide, *K*_i_=5.0±0.2 μm. b) **8ii**: linear mixed inhibition, *K*_i_=3.5±0.4 μm; *K*_i_′=16.8±3.3 μm.

Three of the analogues were chosen for assessment of the mode of inhibition with respect to T[S]_2_, as described in the Experimental Section. Indatraline (**3**), the original indatraline starting point (Figure 2a), is a linear competitive inhibitor of TryR. However, compounds **8ii** (Figure 2b) and **8iii** were confirmed by an *F*-test to show a mode of mixed inhibition. The mixed inhibition mode indicates that these inhibitors are able to bind to the substrate–enzyme complex as well as to the free enzyme, whilst the competitive mode indicates binding only to the free enzyme. However, even for the mixed mode inhibitors, the *K*_i_′ values are approximately fivefold greater than the *K*_i_ values, suggesting that these compounds bind much more favourably to the free enzyme. This subtle shift in mode of inhibition may represent a useful direction for further development, because mixed inhibitors are less susceptible to the effects of substrate accumulation than competitive inhibitors. For these series, driving down the *K*_i_′ value may be more effective than focussing solely on the potency (IC_50_) of the inhibitors.

## Conclusions

We previously reported the screening of the Sigma-LOPAC^1280^ collection of bioactive compounds for inhibitors of TryR and have described follow-up studies on one of the hits we obtained from this collection.^5^, ^7^ Herein we report our attempts to progress a second hit obtained in this screen. Indatraline **3**, a CNS-active, nonselective monoamine reuptake inhibitor, provided the drug-like starting point for our studies. Using a range of synthetic routes, novel indatraline analogues were prepared in order to explore structure–activity relationships. Whilst it proved difficult to significantly increase the potency of the original compound as an inhibitor of TryR, some insight into the preferred substituent on the amine and in the two aromatic rings was deduced. In addition, detailed mode of action studies indicated that two of the inhibitors exhibit a mixed mode of inhibition. This interesting observation has led us to further refine the criteria that need to be considered when developing inhibitors of this important enzyme.

## Experimental Section

### Procedures for the synthesis of analogues of indatraline (3)

Unless otherwise stated, starting materials and reagents were obtained from commercial suppliers and were used without further purification. ^1^H and ^13^C NMR spectra were measured on a Bruker Advance 300/400instrument. Chemical shifts are calibrated with reference to the residual proton and carbon resonances of the solvent (CDCl_3_: *δ*_H_=7.26, *δ*_C_=77.0ppm). Low- and high-resolution mass spectrometric analyses were recorded using chemical ionisation operating in positive or negative ion mode. For assessment of purity of novel compounds that were tested, see table S2 (Supporting Information).^11^

**General procedure for the formation of amines 8 (Method A):** The corresponding amine (0.5mmol) was added to a solution of **7**^11^ (100mg, 0.34mmol) in THF (3mL), and the solution was stirred at 90 °C in a sealed tube for 4h. The solvent was removed in vacuo, and the residue partitioned between H_2_O (10mL) and Et_2_O (10mL). The organic layer was extracted with 10 % citric acid (3×10mL), and the extract was basified with 10 % aqueous NH_4_OH solution then extracted with Et_2_O (3×20mL). The combined organics were dried (Na_2_SO_4_) and concentrated in vacuo. Purification by semi-preparative HPLC (Phenomenex Luna silica column, EtOAc/Hex 6:4 (0.1 % Et_2_NH)) afforded the desired amines.

***Trans*****-[3-(3,4-dichlorophenyl)indan-1-yl]-(3-methylbutyl)amine hydrochloride (8ii):** Yield 72 %, white solid; mp: 190–191 °C; ^1^H NMR (400 MHz, MeOD): *δ*=7.71 (1 H, d, *J*=6.2 Hz, CH_ar_), 7.49 (1 H, d, *J*=8.3 Hz, CH_ar_) 7.45–7.43 (2 H, m, CH_ar_), 7.34 (1 H, d, *J*=1.8 Hz, CH_ar_), 7.12–7.06 (2 H, m, CH_ar_), 4.97–4.95 (1 H, m, CH-N), 4.76 (1 H, t, *J*=7.6 Hz, CH-Ar), 3.17–3.13 (2 H, m, CH_2_N), 2.86–2.81 (1 H, m, CH_*syn*_), 2.53–2.48 (1 H, m, CH_*anti*_), 1.76–1.61 (3 H, m, CH and CH_2_), 0.99 (3 H, d, *J*=6.7 Hz, CH_3_), 0.98 (3 H, d, *J*=6.7 Hz, CH_3_); ^13^C NMR (100 MHz, MeOD): *δ*=148.8 (C), 145.9 (C), 138.4 (C), 133.7 (C), 132.0 (CH ×2), 131.9 (C), 131.2 (CH), 129.3 (CH), 129.1 (CH), 127.3 (CH), 127.0 (CH), 63.3 (CH), 49.4 (CH), 45.8 (CH_2_), 40.3 (CH_2_), 36.1 (CH_2_), 27.3 (CH), 22.7 (CH_3_), 22.6 (CH_3_); LRMS (CI^+^) *m*/*z*: 348.1 [*M*+H]^+^; HRMS (CI^+^) [*M*+H]^+^ *m*/*z* expected for C_20_H_24_N^35^Cl^37^Cl 350.1256, obtained 350.1246.

***Trans*****-[3-(3,4-dichlorophenyl)indan-1-yl]octylamine (8iv):** Yield 60 %, colourless oil; ^1^H NMR (300 MHz, CDCl_3_): *δ*=7.33–7.26 (2 H, m, CH_ar_), 7.20–7.15 (3 H, m, CH_ar_), 6.91–6.88 (2 H, m, CH_ar_), 4.44 (1 H, t, *J*=7.6 Hz, CH-N), 4.28 (1 H, dd, *J*=6.8, 3.4 Hz, CH-Ar), 2.65–2.60 (2 H, m, CH_2_), 2.41–2.32 (1 H, m, CH_*syn*_), 2.21–2.12 (1 H, m, CH_*anti*_), 1.52–1.39 (2 H, m, CH_2_), 1.28–1.12 (10 H, m, CH_2_×5), 0.83–0.79 (3 H, m, CH_3_); ^13^C NMR (100 MHz, CDCl_3_): *δ*=145.7 (C), 145.4 (C), 145.4 (C), 132.5 (C), 130.4 (CH), 130.3 (C), 129.9 (CH), 128.5 (CH), 127.4 (CH), 127.3 (CH), 125.3 (CH), 124.8 (CH), 124.8 (CH), 61.9 (CH), 48.5 (CH), 47.5 (CH_2_), 43.3 (CH_2_), 31.8 (CH_2_), 29.8 (CH_2_), 29.5 (CH_2_), 27.4 (CH_2_), 22.7 (CH_2_), 14.1 (CH_3_); LRMS (CI^+^) *m*/*z*: 390.2 [*M*+H]^+^; HRMS (CI^+^) [*M*+H]^+^ *m*/*z* expected for C_23_H_30_NCl_2_ 390.1755, obtained 390.1755.

***Trans*****-4-[3-(3,4-dichlorophenyl)indan-1-yl]morpholine (8vi):** Yield 61 %, colourless oil; ^1^H NMR (300 MHz, MeOD): *δ*=7.80–7.77 (1 H, m, CH_ar_), 7.52–7.43 (3 H, m, CH_ar_), 7.37 (1 H, d, *J*=1.8 Hz, CH_ar_), 7.12 (1 H, dd, *J*=8.3, 1.8 Hz, CH_ar_), 7.07–7.04 (1 H, m, CH_ar_), 5.10 (1 H, d, *J*=8.0 Hz, CH-N), 4.80 (1 H, t, *J*=8.0 Hz, CH-Ar), 4.09–4.04 (2 H, m, CH_2_), 3.94–3.82 (2 H, m, CH_2_), 3.50–3.45 (1 H, m, CH), 3.37–3.35 (2 H, m, CH_2_), 3.26–3.06 (2 H, m, CH_2_, CH_*syn*_), 2.56–2.45 (1 H, m, CH_*anti*_); ^13^C NMR (75 MHz, MeOD): *δ*=150.3 (C), 145.8 (C), 135.6 (C), 133.7 (C), 132.6 (CH), 132.0 (CH), 131.4 (CH), 129.3 (CH), 129.2 (CH), 128.7 (CH), 127.1 (CH), 72.0 (CH), 65.2 (CH_2_), 65.0 (CH_2_), 50.8 (CH_2_), 50.3 (CH_2_), 50.1 (CH), 38.3 (CH_2_); LRMS (CI^+^) *m*/*z*: 348.1 [*M*+H]^+^; HRMS (CI^+^) [*M*+H]^+^ *m*/*z* expected for C_19_H_20_NOCl_2_ 348.0922, obtained 348.0900.

**General procedure for chalcone 13a–i formation:** A solution of KOH (6mmol) in H_2_O (3mL) was slowly added to a solution of ketone **11** (2mmol) and aldehyde **12** (2mmol) in EtOH (6mL) at 0 °C. The reaction mixture was stirred for 2h, and the resulting precipitate was collected by filtration, washed with EtOH (3mL), and dried in vacuo. See Supporting Information for analytical data for **13a**–**i**.

**General procedure for the Nazarov reaction (4b,f–i):** A solution of **13** (1mmol) in TFA (4mL) was heated in a sealed 10mL tube in a multimode CEM Discover™ microwave at 120 °C for 10min. The powermax mode was enabled, and the microwave was used at its maximum power of 300 W until the set temperature was reached. The solvent was removed in vacuo, and the residue was poured onto ice water and extracted with EtOAc (3×10mL). The combined extracts were washed with saturated NaHCO_3_ solution (20mL) and brine (20mL), dried (Na_2_SO_4_) and reduced in vacuo. Purification through a plug of silica (Hex/EtOAc 20:1) afforded the desired indanone. See Supporting Information for analytical data for **4b,f**–**i**.

**General procedure for the formation of alcohols (6b,f–i):** NaBH_4_ (0.6mmol) was added to a solution of **4** (0.6mmol) in MeOH (3mL), and the reaction was stirred at room temperature for 2h. A 2 m solution of aqueous KOH (3mL) was added, and the reaction mixture was extracted with CH_2_Cl_2_ (3×7mL). The combined organic layers were dried (Na_2_SO_4_) and reduced in vacuo. Purification of the residue through a plug of silica (Hex/EtOAc 10:1) afforded the desired alcohol. See Supporting Information for analytical data for **6b,f**–**i**.

**General procedure for the formation of azides 9b,f–i:** Diphenylphosphoryl azide (0.48mmol) was added to a solution of **6** (0.4mmol) in anhydrous THF (2mL) at 0 °C, and the reaction was stirred for 10min. DBU (0.48mmol) was slowly added, and the reaction mixture was stirred overnight. H_2_O (3mL) was added, and the reaction mixture was extracted with CH_2_Cl_2_ (3×7mL). The combined organic layers were dried (Na_2_SO_4_) and reduced in vacuo. Purification of the residue through a plug of silica (Hex/EtOAc 40:1) afforded the desired azide. See Supporting Information for analytical data for **9b,f**–**i**.

**General procedure for Staudinger reduction to form 10b,f–i:** PS–PPh_3_ (0.6mmol) was added to a solution of **9** (0.3mmol) in anhydrous THF (5mL), and the reaction was stirred for 16h. H_2_O (1mL) was added, and the reaction was stirred for a further 4h. The reaction mixture was filtered and extracted with CH_2_Cl_2_ (3×5mL). The combined organic layers were dried (Na_2_SO_4_) and concentrated in vacuo to give the desired amine **10**. See Supporting Information for analytical data for **10b,f**–**i**.

**General procedure for reductive amination of 10:** The corresponding aldehyde (0.15mmol) was added to a solution of **10** (0.18mmol) in anhydrous THF (1mL), and the solution was stirred for 1h. Sodium triacetoxyborohydride (0.36mmol) was added, and the mixture was stirred overnight. A 2 m solution of aqueous KOH (1mL) was added, and the reaction was extracted with CH_2_Cl_2_ (3×1mL). The combined organics were dried (Na_2_SO_4_) and filtered. PS–benzaldehyde resin (0.1mmol) was added, and the mixture was agitated for 2h. The resin was removed by filtration, and the solvent was concentrated in vacuo to afford the desired amines.

***Trans*****-[3-(3,4-dichlorophenyl)indan-1-yl]-(4-chlorobenzyl)amine (8viii):** Yield 54 %, colourless oil; ^1^H NMR (300 MHz, CDCl_3_): *δ*=7.35–7.14 (9 H, m, CH_ar_), 6.93–6.86 (2 H, m, CH_ar_), 4.46 (1 H, t, *J*=7.5 Hz, CH-N), 4.32 (1 H, dd, *J*=6.8, 3.5 Hz, CH-Ar), 3.79 (2 H, s, CH_2_Ar), 2.44–2.36 (1 H, m, CH_*syn*_), 2.23–2.14 (1 H, m, CH_*anti*_); ^13^C NMR (100 MHz, CDCl_3_): *δ*=145.5 (C), 145.5 (C), 145.1 (C), 138.8 (C), 132.7 (C), 132.5 (C), 130.5 (CH), 130.3 (C), 129.9 (CH), 129.4 (CH ×2), 128.5 (CH ×2), 128.4 (CH), 127.4 (CH), 127.3 (CH), 125.3 (CH), 124.6 (CH), 61.6 (CH), 51.0 (CH_2_), 48.5 (CH), 43.8 (CH_2_); LRMS (CI^+^) *m*/*z*: 402.1 [*M*+H]^+^; HRMS (CI^+^) [*M*+H]^+^ *m*/*z* expected for C_22_H_19_NCl_3_ 402.0583, obtained 402.0573.

***Trans*****-[3-(3,4-dichlorophenyl)indan-1-yl]-(3,4-dichlorobenzyl)amine (8ix):** Yield 73 %, colourless oil; ^1^H NMR (300 MHz, CDCl_3_): *δ*=7.42 (1 H, d, *J*=2.0 Hz, CH_ar_), 7.34 (1 H, dd, *J*=6.4, 2.6 Hz, CH_ar_), 7.31 (1 H, d, *J*=8.2 Hz, CH_ar_), 7.28 (1 H, d, *J*=8.2 Hz, CH_ar_), 7.22–7.18 (2 H, m, CH_ar_), 7.16–7.12 (2 H, m, CH_ar_), 6.94–6.91 (1 H, m, CH_ar_), 6.88 (1 H, dd, *J*=8.2, 2.0 Hz, CH_ar_), 4.46 (1 H, t, *J*=7.5 Hz, CH-N), 4.32 (1 H, dd, *J*=6.7, 3.6 Hz, CH-Ar), 3.78 (2 H, s, CH_2_-Ar), 2.43–2.35 (1 H, m, CH_*syn*_), 2.24–2.15 (1 H, m, CH_*anti*_); ^13^C NMR (100 MHz, CDCl_3_): *δ*=145.4 (C), 145.4 (C), 145.0 (C), 140.8 (C), 132.5 (C), 132.4 (C), 130.8 (C), 130.5 (CH), 130.3 (CH), 129.9 (CH), 129.8 (CH), 129.5 (C), 128.5 (CH), 127.4 (CH), 127.4 (CH), 127.3 (CH), 125.3 (CH), 124.6 (CH), 61.6 (CH), 50.5 (CH_2_), 48.5 (CH), 43.8 (CH_2_); LRMS (CI^+^) *m*/*z*: 436.0 [*M*+H]^+^; HRMS (CI^+^) [*M*+H]^+^ *m*/*z* expected for C_22_H_18_NCl_4_ 436.0193, obtained 436.0194.

***Trans*****-[3-(3,4-dichlorophenyl)indan-1-yl]-(4-methylbenzyl)amine (8x):** Yield 77 %, colourless oil; ^1^H NMR (300 MHz, CDCl_3_): *δ*=7.35–7.34 (1 H, m, CH_ar_), 7.27 (1 H, d, *J*=8.2 Hz, CH_ar_), 7.19–7.14 (5 H, m, CH_ar_), 7.08–7.06 (2 H, m, CH_ar_), 6.92–6.86 (2 H, m, CH_ar_), 4.46 (1 H, t, *J*=7.5 Hz, CH-N), 4.33 (1 H, dd, *J*=6.8, 3.5 Hz, CH-Ar), 3.78 (2 H, s, CH_2_-Ar), 2.45–2.37 (1 H, m, CH_*syn*_), 2.26 (3 H, s, CH_3_), 2.22–2.13 (1 H, m, CH_*anti*_); ^13^C NMR (75 MHz, CDCl_3_): *δ*=145.6 (C), 145.5 (C), 145.5 (C), 137.2 (C), 136.6 (C), 132.4 (C), 130.4 (CH), 130.2 (C), 129.9 (CH), 129.1 (CH ×2), 128.2 (CH), 128.1 (CH ×2), 127.4 (CH), 127.2 (CH), 125.2 (CH), 124.6 (CH), 61.4 (CH), 51.5 (CH_2_), 48.6 (CH), 43.8 (CH_2_), 21.1 (CH_3_); LRMS (CI^+^) *m*/*z*: 382.1 [*M*+H]^+^; HRMS (CI^+^) [*M*+H]^+^ *m*/*z* expected for C_23_H_22_NCl_2_ 382.1129, obtained 382.1134.

***Trans*****-[3-(3,4-dichlorophenyl)indan-1-yl]-(4-methoxybenzyl)amine (8xi):** Yield 83 %, colourless oil; ^1^H NMR (300 MHz, CDCl_3_): *δ*=7.42–7.39 (1 H, m, CH_ar_), 7.35 (1 H, d, *J*=8.3 Hz, CH_ar_), 7.30–7.22 (5 H, m, CH_ar_), 6.99–6.94 (2 H, m, CH_ar_), 6.91–6.85 (2 H, m, CH_ar_), 4.54 (1 H, t, *J*=7.7 Hz, CH-N), 4.41 (1 H, dd, *J*=6.8, 3.5 Hz, CH-Ar), 3.84 (2 H, s, CH_2_-Ar), 3.80 (3 H, s, CH_3_), 2.49 (1 H, ddd, *J*=13.1, 7.7, 3.5 Hz, CH_*syn*_), 2.25 (1 H, dt, *J*=13.1, 6.8 Hz, CH_*anti*_), 1.69 (1 H, br s, NH); ^13^C NMR (75 MHz, CDCl_3_): *δ*=158.7 (C), 145.6 (C), 145.5 (C), 145.5 (C), 145.3 (C), 132.4 (C), 130.4 (CH), 130.2 (C), 129.9 (CH), 129.3 (CH ×2), 128.2 (CH), 127.4 (CH), 127.2 (CH), 125.2 (CH), 124.6 (CH), 113.8 (CH ×2), 61.5 (CH), 55.3 (CH_3_), 51.2 (CH_2_), 48.6 (CH), 43.6 (CH_2_); LRMS (CI^+^) *m*/*z*: 398.1 [*M*+H]^+^; HRMS (CI^+^) [*M*+H]^+^ *m*/*z* expected for C_23_H_22_NOCl_2_ 398.1078, obtained 398.1082.

***Trans*****-[3-(3,4-dichlorophenyl)indan-1-yl]-(4-dimethylaminobenzyl)amine (8xii):** Yield 68 %, colourless oil; ^1^H NMR (300 MHz, CDCl_3_): *δ*=7.46–7.43 (1 H, m, CH_ar_), 7.38 (1 H, d, *J*=8.3 Hz, CH_ar_), 7.32–7.24 (5 H, m, CH_ar_), 7.02–6.98 (2 H, m, CH_ar_), 6.76 (2 H, d, *J*=8.5 Hz, CH_ar_), 4.58 (1 H, t, *J*=7.7 Hz, CH-N), 4.46 (1 H, dd, *J*=6.9, 3.3 Hz, CH-Ar), 3.84 (2 H, s, CH_2_-Ar), 2.97 (6 H, s, CH_3_ ×2), 2.53 (1 H, ddd, *J*=13.0, 7.7, 3.3 Hz, CH_*syn*_), 2.28 (1 H, dt, *J*=13.0, 6.9 Hz, CH_*anti*_), 2.15 (1 H, br s, NH); ^13^C NMR (75 MHz, CDCl_3_): *δ*=149.8 (C), 145.6 (C), 145.5 (C), 145.3 (C), 132.4 (C), 130.4 (CH), 130.1 (C), 129.9 (CH), 129.1 (CH ×2), 128.1 (CH), 128.0 (C), 127.4 (CH), 127.2 (CH), 125.1 (CH), 124.6 (CH), 112.7 (CH ×2), 61.3 (CH), 51.3 (CH_2_), 48.5 (CH), 43.7 (CH_2_), 40.9 (CH_3_ ×2); LRMS (CI^+^) *m*/*z*: 411.1 [*M*+H]^+^; HRMS (CI^+^) [*M*+H]^+^ *m*/*z* expected for C_24_H_25_N_2_Cl_2_ 411.1395, obtained 411.1385.

***Trans*****-[3-(3,4-dichlorophenyl)indan-1-yl]-(4-aminobenzyl)amine (8xiii):** Yield 76 %, colourless oil; ^1^H NMR (400 MHz, CDCl_3_): *δ*=7.41–7.39 (1 H, m, CH_ar_), 7.34 (1 H, d, *J*=8.3 Hz, CH_ar_), 7.26–7.21 (3 H, m, CH_ar_), 7.15 (2 H, d, *J*=8.4 Hz, CH_ar_), 6.98–6.95 (2 H, m, CH_ar_), 6.66 (2 H, d, *J*=8.4 Hz, CH_ar_), 4.54 (1 H, t, *J*=7.8 Hz, CH-N), 4.41 (1 H, dd, *J*=6.9, 3.3 Hz, CH-Ar), 3.78 (2 H, d, *J*=3.0 Hz, CH_2_-Ar), 2.49 (1 H, ddd, *J*=13.2, 7.8, 3.3 Hz, CH_*syn*_), 2.24 (1 H, dt, *J*=13.2, 6.9 Hz, CH_*anti*_), 1.75 (3 H, br s, NH ×3); ^13^C NMR (100 MHz, CDCl_3_): *δ*=149.6 (C), 145.6 (C), 145.5 (C), 145.3 (C), 132.4 (C), 130.4 (CH), 130.1 (C), 129.9 (CH), 129.3 (CH ×2), 128.2 (CH), 128.0 (C), 127.4 (CH), 127.2 (CH), 125.2 (CH), 124.6 (CH), 115.2 (CH ×2), 61.3 (CH), 51.3 (CH_2_), 48.6 (CH), 43.7 (CH_2_); LRMS (CI^+^) *m*/*z*: 383.1 [*M*+H]^+^; HRMS (CI^+^) [*M*+H]^+^ *m*/*z* expected for C_22_H_21_N_2_Cl_2_ 383.1082, obtained 383.1084.

***Trans*****-*N*-[3-(3,4-dichlorophenyl)indan-1-yl]acetamide (8xiv):** Acetyl chloride (15.6 μL, 0.22mmol) and DIPEA (115 μL, 0.66mmol) were added to a solution of **10a** (50mg, 0.18mmol) in anhydrous THF (1mL), and the solution was stirred overnight. A saturated aqueous solution of NaHCO_3_ (1mL) was added, and the reaction was extracted with CH_2_Cl_2_ (3×1mL). The combined organics were dried (Na_2_SO_4_) and concentrated in vacuo. Purification through a plug of silica (Hex/EtOAc 5:1) afforded **8xiv** as a white solid (84 %); mp: 127–128 °C; ^1^H NMR (300 MHz, CDCl_3_): *δ*=7.42–7.39 (1 H, m, CH_ar_), 7.35 (1 H, d, *J*=8.2 Hz, CH_ar_), 7.32–7.28 (2 H, m, CH_ar_), 7.19 (1 H, d, *J*=2.1 Hz, CH_ar_), 7.05–7.02 (1 H, m, CH_ar_), 6.92 (1 H, dd, *J*=8.3, 2.1 Hz, CH_ar_), 5.67–5.56 (2 H, m, NH, CH-N), 4.46 (1 H, t, *J*=7.2 Hz, CH-Ar), 2.52–2.42 (2 H, m, CH_2_), 2.03 (3 H, s, CH_3_); LRMS (CI^+^) *m*/*z*: 320.1 [*M*+H]^+^; HRMS (CI^+^) [*M*+H]^+^ *m*/*z* expected for C_17_H_16_Cl_2_NO 320.0609, obtained 320.0616.

***Trans*****-*N*-[3-(3,4-dichlorophenyl)indan-1-yl]-4-methylbenzenesulfonamide (8xv):** *p*-Toluenesulfonyl chloride (42mg, 0.22mmol) and DIPEA (115 μL, 0.66mmol) were added to a solution of **10a** (50mg, 0.18mmol) in anhydrous THF (1mL), and the solution was stirred overnight. A saturated aqueous solution of NaHCO_3_ was added, and the reaction was extracted with CH_2_Cl_2_ (3×1mL). The combined organics were dried (Na_2_SO_4_) and concentrated in vacuo. Purification through a plug of silica (Hex/EtOAc 5:1) afforded **8xv** as a white solid (77 %); mp: 149–150 °C; ^1^H NMR (300 MHz, CDCl_3_): *δ*=7.82 (2 H, d, *J*=8.3 Hz, CH_ar_), 7.36–7.31 (3 H, m, CH_ar_), 7.26–7.23 (2 H, m, CH_ar_), 7.13–7.10 (2 H, m, CH_ar_), 7.00–6.97 (1 H, m, CH_ar_), 6.85 (1 H, d, *J*=8.3, 2.1 Hz, CH_ar_), 4.97–4.91 (1 H, m, CH-N), 4.65 (1 H, d, *J*=8.0 Hz, NH), 4.44–4.39 (1 H, m, CH-Ar), 2.46 (3 H, s, CH_3_), 2.44–2.38 (1 H, m, CH_*syn*_), 2.27–2.18 (1 H, m, CH_*anti*_); ^13^C NMR (75 MHz, CDCl_3_): *δ*=144.9 (C), 144.4 (C), 143.7 (C), 141.9 (C), 137.8 (C), 134.2 (C), 132.6 (C), 130.6 (CH), 129.9 (CH ×2), 129.6 (CH), 129.5 (CH), 129.3 (CH), 128.1 (CH), 127.1 (CH ×2), 125.3 (CH), 124.7 (CH), 57.7 (CH), 47.9 (CH), 44.1 (CH_2_), 21.6 (CH_3_); LRMS (CI^+^) *m*/*z*: 432.1 [*M*+H]^+^; HRMS (CI^+^) [*M*+H]^+^ *m*/*z* expected for C_22_H_20_NO_2_Cl_2_S 432.0592, obtained 432.0590.

***Trans*****-[3-(3,4-dichlorophenyl)-6-methoxyindan-1-yl]-(3-methylbutyl)amine (8xvi):** Yield (52 %), colourless oil; ^1^H NMR (300 MHz, CDCl_3_): *δ*=7.28 (1 H, d, *J*=8.3 Hz, CH_ar_), 7.13 (1 H, d, *J*=2.2 Hz, CH_ar_), 6.90–6.80 (3 H, m, CH_ar_), 6.71 (1 H, dd, *J*=8.3, 2.2 Hz, CH_ar_), 4.36 (1 H, t, *J*=7.3 Hz, CH-N), 4.25 (1 H, dd, *J*=6.8, 4.0 Hz, CH-Ar), 3.75 (3 H, s, CH_3_), 2.67–2.62 (2 H, m, CH_2_), 2.40–2.32 (1 H, m, CH_syn_), 2.23–2.14 (1 H, m, CH_anti_), 1.58 (1 H, sept, *J*=6.6 Hz, CH), 1.38–1.31 (2 H, m, CH_2_), 0.84 (3 H, d, *J*=6.6 Hz, CH_3_), 0.83 (3 H, d, *J*=6.6 Hz, CH_3_); ^13^C NMR (75 MHz, CDCl_3_): *δ*=159.5 (C), 144.3 (C), 139.1 (C), 137.4 (C), 132.7 (C), 130.9 (C), 130.6 (CH), 129.9 (CH), 127.3 (CH), 126.3 (CH), 117.9 (CH), 110.9 (CH), 60.9 (CH), 55.7 (CH_2_), 48.0 (CH), 42.8 (CH_2_), 39.4 (CH_2_), 34.4 (CH_2_), 26.2 (CH), 22.3 (CH_3_), 22.1 (CH_3_); LRMS (CI^+^) *m*/*z*: 378.1 [*M*+H]^+^; HRMS (CI^+^) [*M*+H]^+^ *m*/*z* expected for C_21_H_26_Cl_2_NO 378.1391, obtained 378.1399.

***Trans*****-[3-(3,4-dibromophenyl)-6-methoxyindan-1-yl]-(3-methylbutyl)amine (8xvii):** Yield (71 %), colourless oil; ^1^H NMR (300 MHz, CDCl_3_): *δ*=7.50 (1 H, d, *J*=8.2 Hz, CH_ar_), 7.38 (1 H, d, *J*=2.0 Hz, CH_ar_), 6.93–6.86 (3 H, m, CH_ar_), 6.79 (1 H, dd, *J*=8.4, 2.5 Hz, CH_ar_), 4.42 (1 H, t, *J*=7.5 Hz, CH-N), 4.31 (1 H, dd, *J*=6.8, 3.6 Hz, CH-Ar), 3.82 (3 H, s, CH_3_), 2.73–2.68 (2 H, m, CH_2_), 2.47–2.39 (1 H, m, CH_syn_), 2.29–2.21 (1 H, m, CH_anti_), 1.64 (1 H, sept, *J*=6.6 Hz, CH), 1.45–1.37 (2 H, m, CH_2_), 0.91 (3 H, d, *J*=6.6 Hz, CH_3_), 0.90 (3 H, d, *J*=6.6 Hz, CH_3_); LRMS (CI^+^) *m*/*z*: 468.0 [*M*+H]^+^; HRMS (CI^+^) [*M*+H]^+^ *m*/*z* expected for C_21_H_26_NO^79^Br^81^Br 468.0361, obtained 468.0356.

***Trans*****-[3-(4-chlorophenyl)-6-methoxyindan-1-yl]-(3-methylbutyl)amine (8xviii):** Yield (56 %), colourless oil; ^1^H NMR (300 MHz, CDCl_3_): *δ*=7.24–7.22 (2 H, m, CH_ar_), 7.05–6.86 (4 H, m, CH_ar_), 6.77 (1 H, dd, *J*=8.3, 2.6 Hz, CH_ar_), 4.45 (1 H, t, *J*=7.3 Hz, CH-N), 4.31 (1 H, dd, *J*=7.1, 3.8 Hz, CH-Ar), 3.82 (3 H, s, CH_3_), 2.72–2.69 (2 H, m, CH_2_), 2.47–2.38 (1 H, m, CH_syn_), 2.31–2.22 (1 H, m, CH_anti_), 1.62 (1 H, sept, *J*=6.6 Hz, CH), 1.45–1.38 (2 H, m, CH_2_), 0.91 (3 H, d, *J*=6.6 Hz, CH_3_), 0.90 (3 H, d, *J*=6.6 Hz, CH_3_); LRMS (CI^+^) *m*/*z*: 344.2 [*M*+H]^+^; HRMS (CI^+^) [*M*+H]^+^ *m*/*z* expected for C_21_H_27_NOCl 344.1781, obtained 344.1784.

***Trans*****-[3-(4-methylphenyl)-6-methoxyindan-1-yl]-(3-methylbutyl)amine (8xix):** Yield (73 %), colourless oil; ^1^H NMR (300 MHz, CDCl_3_): *δ*=7.05 (2 H, d, *J*=8.0 Hz, CH_ar_), 7.01 (2 H, d, *J*=8.0 Hz, CH_ar_), 6.97–6.89 (2 H, m, CH_ar_), 6.76 (1 H, dd, *J*=8.2, 2.4 Hz, CH_ar_), 4.44 (1 H, t, *J*=7.4 Hz, CH-N), 4.32 (1 H, dd, *J*=6.7, 3.7 Hz, CH-Ar), 3.82 (3 H, s, CH_3_), 2.74–2.70 (2 H, m, CH_2_), 2.45–2.39 (1 H, m, CH_syn_), 2.31–2.24 (4 H, m, CH_3_, CH_anti_), 1.64 (1 H, sept, *J*=6.6 Hz, CH), 1.44–1.39 (2 H, m, CH_2_), 0.91 (3 H, d, *J*=6.6 Hz, CH_3_), 0.90 (3 H, d, *J*=6.6 Hz, CH_3_); LRMS (CI^+^) *m*/*z*: 324.2 [*M*+H]^+^; HRMS (CI^+^) [*M*+H]^+^ *m*/*z* expected for C_22_H_30_NO 324.2327, obtained 324.2332.

***Trans*****-[3-(4-methoxyphenyl)-6-methoxyindan-1-yl]-(3-methylbutyl)amine (8xx):** Yield (76 %), colourless oil; ^1^H NMR (300 MHz, CDCl_3_): *δ*=7.22–7.16 (1 H, m, CH_ar_), 6.94–6.91 (2 H, m, CH_ar_), 6.78–6.61 (4 H, m, CH_ar_), 4.45 (1 H, t, *J*=7.4 Hz, CH-N), 4.33 (1 H, dd, *J*=6.7, 3.7 Hz, CH-Ar), 3.82 (3 H, s, CH_3_), 3.75 (3 H, s, CH_3_), 2.74–2.70 (2 H, m, CH_2_), 2.46–2.39 (1 H, m, CH_syn_), 2.31–2.26 (1 H, m, CH_anti_), 1.64 (1 H, sept, *J*=6.6 Hz, CH), 1.44–1.39 (2 H, m, CH_2_), 0.91 (3 H, d, *J*=6.6 Hz, CH_3_), 0.90 (3 H, d, *J*=6.6 Hz, CH_3_); LRMS (CI^+^) *m*/*z*: 340.2 [*M*+H]^+^; HRMS (CI^+^) [*M*+H]^+^ *m*/*z* expected for C_22_H_30_NO_2_ 340.2277, obtained 340.2276.

***Trans*****-benzyl-[3-(3,4-dichlorophenyl)-6-methoxyindan-1-yl]amine (8xxi):** Yield (59 %), colourless oil; ^1^H NMR (300 MHz, CDCl_3_): *δ*=7.32–7.12 (7 H, m, CH_ar_), 6.89–6.81 (3 H, m, CH_ar_), 6.72 (1 H, dd, *J*=8.3, 2.5 Hz, CH_ar_), 4.39 (1 H, t, *J*=7.3 Hz, CH-N), 4.31 (1 H, dd, *J*=6.8, 4.0 Hz, CH-Ar), 3.82 (2 H, s, CH_2_-Ph), 3.75 (3 H, s, CH_3_), 2.46–2.37 (1 H, m, CH_syn_), 2.24–2.15 (1 H, m, CH_anti_); ^13^C NMR (75 MHz, CDCl_3_): *δ*=159.5 (C), 144.4 (C), 144.4 (C), 139.2 (C), 137.5 (C), 132.6 (C), 130.6 (CH), 130.5 (CH ×2), 130.1 (C), 129.9 (CH), 129.1 (CH ×2), 127.4 (CH), 126.3 (CH), 117.9 (CH), 110.8 (CH), 59.7 (CH), 55.7 (CH_3_), 47.9 (CH), 47.7 (CH_2_), 39.1 (CH_2_); LRMS (CI^+^) *m*/*z*: 398.1 [*M*+H]^+^; HRMS (CI^+^) [*M*+H]^+^ *m*/*z* expected for C_23_H_22_NOCl_2_ 398.1078, obtained 398.1090.

**Inhibition of trypanothione reductase:** Compounds were tested for inhibition of *T. cruzi* TryR by using a high-throughput microplate assay.^15^ Briefly, assays were set up in 96-well plates using a Biotek Precision 2000automated liquid handler and initiated with NADPH. The final assay mixtures (0.18mL) contained TryR (20mU mL^−1^), 40mm HEPES (pH 7.5), 1mm EDTA, 0.15mm NADPH, 50 μm DTNB, 6 μm T[S]_2_, and inhibitor (100 μm–5nm in threefold serial dilutions). The rate of TNB^−^ formation was monitored over 5min in a Spectramax 340PC plate reader (Molecular Devices) at *λ*=412nm. Raw data were processed with Microsoft Excel. GraFit 5.0 (Erithacus software) was used to fit the data to a three-parameter equation and output the concentration resulting in 50 % inhibition (IC_50_ value). Compounds were tested against TryR on three separate occasions, and the IC_50_ values were used to calculate a mean weighted to the standard error. These values are given in Table 1. The mean *Z*′ value throughout the IC_50_ testing was 0.88, indicating suitable assay performance.

**Inhibition of glutathione reductase:** Compounds were tested for inhibition of human GR by using a modification of the TryR assay method and processed as before. The final conditions in the assay were 7 μm glutathione disulfide, 150 μm NADPH, 50 μm DTNB, and 16nm human GR.

**Assessment of mode of inhibition:** Three of the compounds (**3**, **8ii**, and **8iii**) were tested for mode of inhibition with respect to trypanothione. The standard TryR assay was used as before. Aliquots of the assay mixture (180 μL) containing three different concentrations of test compound were added to three rows of a microtitre plate, a fourth row contained only the assay mixture. T[S]_2_ was serially diluted across a fifth row of the plate to produce a 12-point range from 500–5.8 μm. The assay was initiated by transferring 20 μL of T[S]_2_ row to each of the assay rows. The final 200 μL assay contained 150 μm NADPH, 50 μm DTNB, and 20mU mL^−1^ TryR and 50–0.58 μm T[S]_2_. The final inhibitor concentration in the three rows of the plate ranged from 0.5× to 3× IC_50_. The rate of reaction was measured as before. Each data set was fitted by nonlinear regression to the Michaelis–Menten equation using GraFit 5.0 (Erithacus software). The resulting individual fits were examined as Lineweaver–Burk transformations, and the graphs were inspected for diagnostic inhibition patterns. The entire dataset was then globally fitted to the appropriate equation (competitive, mixed, or uncompetitive inhibition).

**Inhibition of** ***T. brucei*** **cell proliferation:** Compounds were tested for inhibition of proliferation of whole *T. brucei* bloodstream form cells using a microplate assay as previously described.^16^ Cells were cultured in modified HMI-9media containing 10 % FBS and 10 % Serum Plus (HMI-9T), in which 0.2mm 2-mercaptoethanol was replaced with 0.056mm thioglycerol.^17^ Briefly, test compounds (10mm in DMSO) were serially diluted in medium in 96-well plates (Greiner). Bloodstream *T. brucei* (S427) cells were added to give a final density of 10^3^ cells mL^−1^ in 0.5 % DMSO with inhibitor ranging from 50to 0.2 μm in a total volume of 200 μL. Pentamidine (250–1nm) was included as a standard drug control on each test plate. Plates were incubated at 37 °C in a 5 % CO_2_ humidified atmosphere for 72h, then 45 μm resazurin (Sigma) was added. After incubation for 4–5h, fluorescence due to formation of resorufin was measured at *λ*_ex_=528nm, *λ*_em_=590nm. Compounds were tested against *T. brucei* cells on three separate occasions, and the EC_50_ values were used to calculate a mean weighted to the standard error. These values are given in Table 1. The mean *Z*′ value throughout the *T. brucei* testing was 0.67, indicating suitable assay performance.

## References

[1] Stuart K, Brun R, Croft S, Fairlamb AH, Gurtler RE, McKerrow J, Reed S, Tarleton R (2008). J. Clin. Invest..

[2a] Fairlamb AH, Blackburn P, Ulrich P, Chait BT, Cerami A (1985). Science.

[2b] Fairlamb AH, Cerami A (1992). Annu. Rev. Microbiol..

[3] Bailey S, Smith K, Fairlamb AH, Hunter WS (1993). Eur. J. Biochem..

[4] Krieger S, Schwarz W, Ariyanayagam MR, Fairlamb AH, Krauth-Siegel RL, Clayton C (2002). Mol. Microbiol..

[5] Richardson JL, Nett IRE, Jones DC, Abdille MH, Gilbert IH, Fairlamb AH (2009). ChemMedChem.

[6] Chong CR, Sullivan DJ (2007). Nature.

[7] Patterson S, Jones DC, Shanks EJ, Frearson JA, Gilbert IH, Wyatt PG, Fairlamb AH (2009). ChemMedChem.

[8a] Bøgesø K, Christensen AV, Hyttel J, Liljefors T (1985). J. Med. Chem..

[8b] Hyttel J, Larsen JJ (1985). J. Neurochem..

[8c] Negus SS, Brandt MR, Mello NK (1999). J. Pharmacol. Exp. Ther..

[9] Lipinski CA, Lombardo F, Dominy BW, Feeney PJ (1997). Adv. Drug Delivery Rev..

[10a] Bøgesø KPJ (1983). J. Med. Chem..

[10b] Froimowitz M, Wu K-M, Moussa A, Haidar RM, Jurayj J, George C, Gardner EL (2000). J. Med. Chem..

[12] Gu X-H, Yu H, Jacobson AE, Rothman RB, Dersch CM, George C, Flippen-Anderson JL, Rice KC (2000). J. Med. Chem..

[13] Yu H, Kim IJ, Folk JE, Tian X, Rothman RB, Baumann MH, Dersch CM, Flippen-Andersen JL, Parrish D, Jacobsen AE, Rice KC (2004). J. Med. Chem..

[14a] Topliss JG (1972). J. Med. Chem..

[14b] Topliss JG (1977). J. Med. Chem..

[15] Hamilton CJ, Saravanamuthu A, Eggleston IM, Fairlamb AH (2003). Biochem. J..

[16a] Jones DC, Hallyburton I, Stojanovski L, Read KD, Frearson JA, Fairlamb AH (2010). Biochem. Pharmacol..

[16b] Raz B, Iten M, Grether-Buhler Y, Kaminsky R, Brun R (1997). Acta Trop..

[17a] Hirumi H, Hirumi K (1989). J. Parasitol..

[17b] Greig N, Wyllie S, Patterson S, Fairlamb AH (2009). FEBS J..

