# Supplementary material for: Synthesis and Evaluation of Indatraline-Based Inhibitors for Trypanothione Reductase
Source: ChemMedChem. 2010 Dec 15;6(2):321–8. doi: 10.1002/cmdc.201000442 (PMC3047706; doi:10.1002/cmdc.201000442)
Supplement: Supplementary file 1 [file cmdc0006-0321-SD1.pdf]

CHEMMEDCHEM
Chemistry & Drug Discovery

Supporting Information

© Copyright Wiley-VCH Verlag GmbH & Co. KGaA, 69451 Weinheim, 2011

Synthesis and Evaluation of Indatraline-Based Inhibitors for Trypanothione Reductase

Jeffrey G. A. Walton,[a] Deuan C. Jones,[b] Paula Kiuru,[a] Alastair J. Durie,[a] Nicholas J. Westwood,*[a] and Alan H. Fairlamb*[b]

cmdc_201000442_sm_miscellaneous_information.pdf

Analytical data for additional compounds

General procedure for chalcone 13a-i formation To a solution of ketone **11** (2 mmol) and aldehyde **12** (2 mmol) in EtOH (6 mL) was slowly added a solution of KOH (6 mmol) in water (3 mL) at 0ºC. The reaction mixture was stirred for 2 h and the resulting precipitate was collected *via* filtration, washed with EtOH (3 mL) and dried in vacuo.

The following known compounds were prepared using this procedure:
13a[S1], **13b**[S2]**, 13c**[S3], **13d**[S4], **13g**[S5a-5f], **13h**[S5a], **13i**[S6]

3-(3,4-Dichloro-phenyl)-1-(3-bromo-phenyl)-propenone (13e)
Yield 71%, yellow solid; ^{1}H NMR (400 MHz, $CDCl_3$) δ 8.13 (1H, t, *J* 1.8Hz, CH_{ar}), 7.93 (1H, dt, *J* 7.4, 1.8 Hz, CH_{ar}), 7.74-7.68 (3H, m, CH_{ar}, CH_{alkene}), 7.52-7.38 (4H, m, CH_{ar}, CH_{alkene}); ^{13}C NMR (100 MHz, $CDCl_3$) δ 188.4 (C=O), 142.8 (CH), 142.7 (C), 139.6 (C), 136.0 (CH), 134.7 (C), 133.5 (C), 131.5 (CH), 131.1 (CH), 130.3 (CH), 129.9 (CH), 127.6 (CH), 127.0 (CH), 123.1 (C), 122.8 (CH); LRMS (CI^+) *m/z* 354.9 $[M+H]^+$; HRMS (CI^+) $[M+H]^+$ *m/z* expected for $C_{15}H_{10}O^{79}BrCl_2$ 354.9292, obtained 354.9286.

3-(3,4-Dibromo-phenyl)-1-(3-methoxy-phenyl)-propenone (13f)
Yield 87%, off-white solid; ^{1}H NMR (400 MHz, $CDCl_3$) δ 7.87 (1H, d, *J* 2.0 Hz, CH_{ar}), 7.69-7.51 (5H, m, CH_{ar}, CH_{alkene}), 7.46-7.39 (2H, m, CH_{ar}), 7.14 (1H, ddd, *J* 8.2, 2.7, 0.9 Hz, CH_{ar}), 3.88 (3H, s, CH_3); LRMS (CI^+) *m/z* 394.9 $[M+H]^+$; HRMS (CI^+) $[M+H]^+$ *m/z* expected for $C_{16}H_{13}O_2{}^{79}Br_2$ 394.9282, obtained 394.9281.

General procedure for the Nazarov reaction (4b,f-i) A solution of **13** (1 mmol) in TFA (4 mL) was heat in a microwave at 120ºC for 10 min. The solvent was removed in vacuo and the residue poured onto iced water and extracted with EtOAc (3 x 10 mL). The combined extracts were washed with saturated $NaHCO_3$ solution (20 mL) and brine (20 mL), dried (Na_2SO_4) and reduced in vacuo. Purification through a plug of silica (Hex:EtOAc 20:1) afforded the desired indanone.

The following known compounds were prepared using this procedure:
4a[S7,S8], **4b**[S9,S10,S11]**, 4g**[S5a], **4h**[S5a], **4i**[S12]

3-(3,4-Dibromo-phenyl)-6-methoxy-indan-1-one (4f)
Yield 79%, colourless oil; ^{1}H NMR (300 MHz, $CDCl_3$) δ 7.53 (1H, d, *J* 8.2 Hz, CH_{ar}), 7.38 (1H, d, *J* 2.1 Hz, CH_{ar}), 7.23-7.12 (3H, m, CH_{ar}), 6.90 (1H, dd, *J* 8.2, 2.1 Hz, CH_{ar}), 4.46 (1H, dd, *J* 7.9, 3.5`Hz, CH-Ar), 3.86 (3H, s, CH_3), 3.24 (1H, dd, *J* 19.2, 7.9 Hz, CH_{syn}), 2.61 (1H, dd, *J* 19.2, 3.6 Hz, CH_{anti}); LRMS (CI^+) *m/z* 394.9 $[M+H]^+$; HRMS (CI^+) $[M+H]^+$ *m/z* expected for $C_{16}H_{13}O_2{}^{79}Br_2$ 394.9282, obtained 394.9277.

General procedure for formation of alcohols (6b,f-i) Sodium borohydride (0.6 mmol) was added to a solution of **4** (0.6 mmol) in MeOH (3 mL) and the reaction was stirred at room temperature for 2 h. 2M aqueous KOH solution (3 mL) was added and the reaction mixture was extracted with DCM (3 x 7 mL). The combined organic layers were dried (Na_2SO_4) and reduced in vacuo. Purification of the residue through a plug of silica (Hex:EtOAc 10:1) afforded the desired alcohol.

The following known compounds were prepared using this procedure:
6a[S11], **4b**[S10,S11]

***Cis*-3-(3,4-dibromo-phenyl)-6-methoxy-indan-1-ol (6f)**
Yield 68%, colourless oil; ^{1}H NMR (300 MHz, $CDCl_3$) δ 7.56-7.50 (2H, m, CH_{ar}), 7.05-7.01 (2H, m, CH_{ar}), 6.84-6.82 (2H, m, CH_{ar}), 5.24 (1H, t, *J* 6.9 Hz, CH-O), 4.08 (1H, t, *J* 8.2 Hz, CH-Ar), 3.83 (3H, s, CH_3), 3.06-2.97 (1H, m, CH_{syn}), 1.91-1.82 (1H, m, CH_{anti}); LRMS (CI^+) *m/z* 396.9 $[M+H]^+$; HRMS (CI^+) $[M+H]^+$ *m/z* expected for $C_{16}H_{15}O_2{}^{79}Br_2$ 396.9439, obtained 396.9445.

***Cis*-3-(4-chloro-phenyl)-6-methoxy-indan-1-ol (6g)**
Yield 85%, colourless oil; ^{1}H NMR (400 MHz, $CDCl_3$) δ 7.29-7.25 (4H, m, CH_{ar}), 7.17-7.15 (1H, m, CH_{ar}), 7.02-7.00 (1H, m, CH_{ar}), 6.82-6.80 (1H, m, CH_{ar}), 5.25 (1H, t, *J* 7.1 Hz, CH-O), 4.12 (1H, t, *J* 8.2 Hz, CH-Ar), 3.83 (3H, s, CH_3), 3.05-2.99 (1H, m, CH_{syn}), 1.92-1.84 (1H, m, CH_{anti}); LRMS (CI^+) *m/z* 275.1 $[M+H]^+$; HRMS (CI^+) $[M+H]^+$ *m/z* expected for $C_{16}H_{16}ClO_2$ 275.0839, obtained 275.0847.

***Cis*-3-(4-methyl-phenyl)-6-methoxy-indan-1-ol (6h)**
Yield 77%, colourless oil; ^{1}H NMR (300 MHz, $CDCl_3$) δ 7.14-7.11 (4H, m, CH_{ar}), 7.01 (1H, d, *J* 2.0 Hz, CH_{ar}), 6.86-6.77 (2H, m, CH_{ar}), 5.23 (1H, dd, *J* 14.6, 7.4Hz, CH-O), 4.11 (1H, t, *J* 8.2, CH-Ar), 3.83 (3H, s, CH_3), 3.06-2.97 (1H, m, CH_{syn}), 2.34 (3H, s, CH_3), 1.96-1.84 (1H, m, CH_{anti}); LRMS (CI^+) *m/z* 255.1 $[M+H]^+$; HRMS (CI^+) $[M+H]^+$ *m/z* expected for $C_{17}H_{19}O_2$ 255.1385, obtained 255.1388.

***Cis*-3-(4-methoxy-phenyl)-6-methoxy-indan-1-ol (6i)**
Yield 73%, colourless oil; ^{1}H NMR (300 MHz, $CDCl_3$) δ 7.24-7.21 (1H, m, CH_{ar}), 7.01 (1H, d, *J* 2.3 Hz, CH_{ar}), 6.90-6.77 (5H, m, CH_{ar}), 5.24 (1H, t, *J* 7.3 Hz, CH-O), 4.12 (1H, t, *J* 8.2 Hz, CH-Ar), 3.83 (3H, s, CH_3), 3.78 (3H, s, CH_3), 3.07-2.98 (1H, m, CH_{syn}), 1.99-1.89 (1H, m, CH_{anti}); LRMS (CI^+) *m/z* 271.1 $[M+H]^+$; HRMS (CI^+) $[M+H]^+$ *m/z* expected for $C_{17}H_{19}O_3$ 271.1334, obtained 271.1327.

General procedure for formation of Azides 9a,b,f-i Diphenylphosphoryl azide (0.48 mmol) was added to a solution of **6** (0.4 mmol) in anhydrous THF (2 mL) at 0ºC and the reaction was stirred for 10 min. DBU (0.48 mmol) was slowly added and the reaction mixture was stirred overnight. H_2O (3 mL) was added and the reaction mixture was extracted with DCM (3 x 7 mL). The combined organic layer were dried (Na_2SO_4) and reduced in vacuo. Purification of the residue through a plug of silica (Hex:EtOAc 40:1) afforded the desired azide.

The following known compound was prepared using this procedure:
9b[S10]

***Trans*-1-azido-3-(3,4-dichloro-phenyl)-indan (9a)**
Yield 93%, red oil; ^{1}H NMR (300 MHz, $CDCl_3$) δ 7.49-7.46 (1H, m, CH_{ar}), 7.40-7.32 (3H, m, CH_{ar}), 7.23 (1H, d, *J* 2.1 Hz, CH_{ar}), 7.03-6.96 (2H, m, CH_{ar}), 5.04 (1H, dd, *J* 6.8, 2.3 Hz, CH-N_3), 4.53 (1H, t, *J* 7.9 Hz), 2.61 (1H, ddd, *J* 13.7, 7.9, 2.3 Hz, CH_{syn}), 2.30 (1H, ddd, *J* 13.7, 7.9, 6.8 Hz, CH_{anti}); ^{13}C NMR (75 MHz, $CDCl_3$) δ 145.8 (C), 144.1 (C), 140.6 (C), 132.7 (C), 130.8 (C), 130.6 (CH), 129.9 (CH), 129.7 (CH), 127.8 (CH), 127.4 (CH), 125.4 (CH), 124.8 (CH), 64.7 (CH), 48.5 (CH), 43.4 (CH_2); LRMS (CI^+) *m/z* 304.0 $[M+H]^+$; HRMS (CI^+) $[M+H]^+$ *m/z* expected for $C_{15}H_{12}Cl_2N_3$ 304.0408, obtained 304.0408.

***Trans*-3-azido-1-(3,4-dibromo-phenyl)-5-methoxy-indan (9f)**
Yield 87%, colourless oil; ^{1}H NMR (400 MHz, $CDCl_3$) δ 7.53 (1H, d, *J* 8.2 Hz, CH_{ar}), 7.39 (1H, d, *J* 2.1 Hz, CH_{ar}), 6.97 (1H, d, *J* 2.1 Hz, CH_{ar}), 6.93 (1H, dd, *J* 8.2, 2.0 Hz, CH_{ar}), 6.90-6.85 (2H, m, CH_{ar}), 4.96 (1H, dd, *J* 6.9, 2.5 Hz, CH-N_3), 4.45 (1H, t, *J* 7.5 Hz, CH-Ar), 3.84 (3H, s, CH_3), 2.61 (1H, ddd, *J* 13.7, 7.5, 2.5 Hz, CH_{syn}), 2.30 (1H,

ddd, *J* 13.7, 7.5, 6.9 Hz, CH_{anti}); LRMS (CI^+) *m/z* 421.9 $[M+H]^+$; HRMS (CI^+) $[M+H]^+$ *m/z* expected for $C_{16}H_{14}N_3O^{79}Br_2$ 421.9504, obtained 421.9510.

***Trans*-3-azido-1-(4-chloro-phenyl)-5-methoxy-indan (9g)**
Yield 74%, colourless oil; ^1H NMR (400 MHz, $CDCl_3$) δ 7.28-7.25 (2H, m, CH_{ar}), 7.06 (2H, d, *J* 8.4Hz, CH_{ar}), 6.97 (1H, d, *J* 2.2 Hz, CH_{ar}), 6.91-6.86 (2H, m, CH_{ar}), 4.96 (1H, dd, *J* 6.9, 2.5 Hz, CH-N_3), 4.48 (1H, t, *J* 7.7 Hz, CH-Ar), 3.84 (3H, s, CH_3), 2.61 (1H, ddd, *J* 13.7, 7.7, 2.5 Hz, CH_{syn}), 2.32 (1H, ddd, *J* 13.7, 7.7, 6.9 Hz, CH_{anti}); LRMS (CI^+) *m/z* 421.9 $[M+H]^+$; HRMS (CI^+) $[M+H]^+$ *m/z* expected for $C_{16}H_{15}ClN_3O$ 300.0904, obtained 300.0905.

***Trans*-3-azido-1-(4-methyl-phenyl)-5-methoxy-indan (9h)**
Yield 87%, colourless oil; ^1H NMR (400 MHz, $CDCl_3$) δ 7.12 (2H, d, *J* 8.0 Hz, CH_{ar}), 7.02 (2H,d, *J* 8.0 Hz, CH_{ar}), 6.97-6.92 (2H, m, CH_{ar}), 6.86 (1H, dd, *J* 8.4, 2.4 Hz, CH_{ar}), 4.96 (1H, dd, *J* 6.9, 2.6 Hz, CH-N_3), 4.47 (1H, t, *J* 7.6 Hz, CH-Ar), 3.84 (3H, s, CH_3), 2.61 (1H, ddd, *J* 13.6, 7.6, 2.6 Hz, CH_{syn}), 2.39-2.32 (4H, m, CH_{anti}, CH_3); LRMS (CI^+) *m/z* 280.1 $[M+H]^+$; HRMS (CI^+) $[M+H]^+$ *m/z* expected for $C_{17}H_{18}N_3O$ 280.1450, obtained 280.1445.

***Trans*-3-azido-1-(4-methoxy-phenyl)-5-methoxy-indan (9i)**
Yield 94%, colourless oil; ^1H NMR (400 MHz, $CDCl_3$) δ 7.22 (1H, t, *J* 7.9 Hz, CH_{ar}), 6.97-6.95 (2H, m, CH_{ar}), 6.87 (1H, dd, *J* 8.2, 2.4 Hz, CH_{ar}), 6.78 (1H, ddd, *J* 8.2, 2.4, 0.9 Hz, CH_{ar}), 6.72 (1H, d, *J* 7.9 Hz, CH_{ar}), 6.68-6.66 (1H, m, CH_{ar}), 4.97 (1H, dd, *J* 6.9, 2.8 Hz, CH-N_3), 4.48 (1H, t, *J* 7.6 Hz, CH-Ar), 3.84 (3H, s, CH_3), 3.77 (3H, s, CH_3), 2.62 (1H, ddd, *J* 13.7, 7.6, 2.8 Hz, CH_{syn}), 2.38 (1H, ddd, *J* 13.7, 7.6, 6.9 Hz, CH_{anti}); LRMS (CI^+) *m/z* 296.1 $[M+H]^+$; HRMS (CI^+) $[M+H]^+$ *m/z* expected for $C_{17}H_{18}N_3O_2$ 296.1399, obtained 296.1404.

General Procedure for Staudinger reduction to form 10a,b,f-I PS-PPh_3 (0.6 mmol) was added to a solution of **9** (0.3 mmol) in anhydrous THF (5 mL) and the reaction was stirred for 16 h. H_2O (1 mL) was added and the reaction was stirred for a further 4 h. The reaction mixture was filtered and extracted with DCM (3 x 5 mL). The combined organic layers were dried (Na_2SO_4) and concentrated in vacuo to give the desired amine **10**. See ESI for analytical data for **10b,f-i**.

The following known compound was also prepared using literature methods:
10a[S13]

***Trans*-3-(3,4-dichloro-phenyl)-6-methoxy-indan-1-ylamine (10b)**
Yield 97%, colourless oil; ^1H NMR (300 MHz, $CDCl_3$) δ 7.33 (1H, d, *J* 8.3 Hz, CH_{ar}), 7.16 (1H, d, *J* 2.1 Hz, CH_{ar}), 6.95-6.91 (3H, m, CH_{ar}), 6.80 (1H, dd, *J* 8.3, 2.4 Hz, CH_{ar}), 4.50 (1H, t, *J* 6.3 Hz, CH-Ar), 4.43 (1H, dd, *J* 8.0, 5.4 Hz, CH-N), 3.83 (3H, s, CH_3), 2.43-2.24 (2H, m, CH_2); LRMS (CI^+) *m/z* 308.1 $[M+H]^+$; HRMS (CI^+) $[M+H]^+$ *m/z* expected for $C_{16}H_{16}Cl_2NO$ 308.0609, obtained 308.0603.

***Trans*-3-(3,4-dibromo-phenyl)-6-methoxy-indan-1-ylamine (10f)**
Yield 95%, colourless oil; ^1H NMR (300 MHz, $CDCl_3$) δ 7.49 (1H, d, *J* 8.2 Hz, CH_{ar}), 7.35 (1H, d, *J* 2.1 Hz, CH_{ar}), 6.94-6.90 (2H, m, CH_{ar}), 6.88 (1H, dd, *J* 8.3, 2.1 Hz, CH_{ar}), 6.80 (1H, dd, *J* 8.2, 2.4 Hz, CH_{ar}), 4.50 (1H, t, *J* 6.2 Hz, CH-Ar), 4.42 (1H, dd, *J* 8.0, 5.5 Hz, CH-N), 3.83 (3H, s, CH_3), 2.43-2.24 (2H, m, CH_2); LRMS (CI^+) *m/z* 396.0 $[M+H]^+$; HRMS (CI^+) $[M+H]^+$ *m/z* expected for $C_{16}H_{16}NO^{79}Br_2$ 395.9599, obtained 395.9599.

***Trans*-3-(4-chloro-phenyl)-6-methoxy-indan-1-ylamine (10g)**

Yield 90%, colourless oil; ^1H NMR (300 MHz, $CDCl_3$) δ 7.24-7.22 (2H, m, CH_{ar}), 7.03-7.00 (2H, m, CH_{ar}), 6.94-6.91 (2H, m, CH_{ar}), 6.79 (1H, dd, *J* 8.3, 2.4 Hz, CH_{ar}), 4.53-4.43 (2H, m, CH-N, CH-Ar), 3.83 (3H, s, CH_3), 2.44-2.25 (2H, m, CH_2); LRMS (CI^+) *m/z* 274.1 $[M+H]^+$; HRMS (CI^+) $[M+H]^+$ *m/z* expected for $C_{16}H_{17}ClNO$ 274.0999, obtained 274.1006.

***Trans*-3-(4-methyl-phenyl)-6-methoxy-indan-1-ylamine (10h)**

Yield 98%, colourless oil; ^1H NMR (300 MHz, $CDCl_3$) δ 7.08 (2H, d, *J* 7.9 Hz, CH_{ar}), 7.00-6.94 (4H, m, CH_{ar}), 6.78 (1H, dd, *J* 8.5, 2.2 Hz, CH_{ar}), 4.52 (1H, t, *J* 6.2 Hz, CH-Ar), 4.45 (1H, dd, *J* 8.0, 5.5 Hz, CH-N), 3.82 (3H, s, CH_3), 2.44 (1H, ddd, *J* 12.6, 7.0, 5.5 Hz, CH_{anti}), 2.31 (4H, m, CH_3, CH_{syn}); LRMS (CI^+) *m/z* 254.2 $[M+H]^+$; HRMS (CI^+) $[M+H]^+$ *m/z* expected for $C_{17}H_{20}NO$ 254.1545, obtained 254.1538.

***Trans*-3-(4-methoxy-phenyl)-6-methoxy-indan-1-ylamine (10i)**

Yield 94%, colourless oil; ^1H NMR (300 MHz, $CDCl_3$) δ 7.19 (1H, t, *J* 7.9 Hz, CH_{ar}), 6.99-6.95 (2H, m, CH_{ar}), 6.80-6.64 (4H, m, CH_{ar}), 4.53 (1H, t, *J* 6.2 Hz, CH-Ar), 4.46 (1H, dd, *J* 8.1, 5.4 Hz, CH-N), 3.82 (3H, s, CH_3), 3.76 (3H, s, CH_3), 2.47 (1H, ddd, 1H, *J* 12.5, 6.2, 5.4 Hz, CH_{anti}), 2.35-2.26 (1H, m, CH_{syn}); LRMS (CI^+) *m/z* 270.2 $[M+H]^+$; HRMS (CI^+) $[M+H]^+$ *m/z* expected for $C_{17}H_{20}NO_2$ 270.1494, obtained 270.1490.

General procedure for reductive amination of 10 To a solution of **10** (0.18 mmol) in anhydrous THF (1 mL) was added the corresponding aldehyde (0.15 mmol) and the solution was stirred for 1 h. Sodium triacetoxyborohydride (0.36 mmol) was added and the mixture was stirred overnight. 2M aqueous KOH solution (1 mL) was added and reaction was extracted with DCM (3 x 1 mL). The combined organics were dried (Na_2SO_4) and filtered. PS-benzaldehyde resin (0.1 mmol) was added and the mixture agitated for 2 h. The resin was removed by filtration and the solvent concentrated in vacuo to afford the desired amines.

The following known compounds were prepared during the course of these studies:
8i[S11], **8iii**[S14], **8v**[S11], **8vii**[S11]

Additional biological data

Table S1. Supplementary Biological data for selected indatraline analogues.

Entry	Compound	R^1	R^2	R^3	R^4	TryR IC_{50} (μM)	*T.brucei* EC_{50} (μM)
1	**S8xxii**	Cyclohexyl	H	H	3,4-Cl_2	6.14 (±0.40)	2.03 (±0.30)
2	**S8xxiii**	*Iso*-butyl	H	H	3,4-Cl_2	6.47 (±0.25)	2.08 (±0.19)
3	**S8xxiv**	*n*-Butyl	H	H	3,4-Cl_2	6.13 (±0.33)	1.60 (±0.16)
4	**S8xxv**	*n*-Pentyl	H	H	3,4-Cl_2	4.61 (±0.44)	1.42 (±0.14)
5	**S8xxvi**	*n*-Hexyl	H	H	3,4-Cl_2	4.96 (±0.33)	1.50 (±0.14)
6	**S8xxvii**	Cyclohexanemethyl	H	H	3,4-Cl_2	4.94 (±0.33)	1.68 (±0.14)
7	**S8xxviii**	2-Naphthylmethyl	H	H	3,4-Cl_2	61.6 (±3.1)	4.68 (±0.49)
8	**S8xxix**	2-Thiophenemethyl	H	H	3,4-Cl_2	15.7 (±2.2)	5.10 (±0.44)
9	**S8xxx**	-$(CH_2)_2NH(CH_2)_2$-.		H	3,4-Cl_2	26.0 (±2.0)	1.77 (±0.19)
10	**S8xxxi**	4-*n*Bu-Bn	H	H	3,4-Cl_2	37.8 (±3.0)	3.22 (±0.12)
11	**S8xxxii**	4-Br-Bn	H	H	3,4-Cl_2	31.4 (±3.5)	3.09 (±0.55)
12	**S8xxxiii**	4-NO_2-Bn	H	H	3,4-Cl_2	31.2 (±3.6)	3.88 (±0.38)
13	**S8xxxiv**	3-MeO-Bn	H	H	3,4-Cl_2	6.62 (±0.77)	3.21 (±0.27)
14	**S8xxxv**	2-MeO-Bn	H	H	3,4-Cl_2	7.17 (±0.75)	1.53 (±0.12)
15	**S8xxxvi**	Benzoyl	H	H	3,4-Cl_2	>200	11.7 (±0.5)
16	**S8xxxvii**	*Iso*-amyl	H	6-MeO	2-MeO	49.4 (±3.7)	N.D. [a]
17	**S8xixxvii**	*Iso*-amyl	H	6-MeO	3-Br	12.6 (±1.0)	2.67 (±0.53)
18	**S8xxxix**	*Iso*-amyl	H	6-MeO	3-NO_2	38.9 (±1.6)	N.D.
19	**S8xxxx**	*Iso*-amyl	H	6-MeO	4-Br	10.7 (±0.9)	2.19 (±0.27)
20	**S8xxxxi**	*Iso*-amyl	H	6-MeO	1-Naphthyl	15.9 (±1.2)	2.13 (±0.17)

[a] Not determined

**Table S2** Assessment of Purity for Novel Compounds in Table 1				
Compound number	Retention Time (min)	% Purity (by HPLC)	Theoretical HRMS	(Experimental – theoretical)
**3** (known)	-	-	-	-
**5** (known)	-	-	292.0660	1.0×10^{-3}
**8i** (known)	-	-	306.0816	-0.8×10^{-3}
**8ii**	2.98[a]	Single peak	350.1256	-1.0×10^{-3}
**8iii** (known)	-	-	334.1129	-0.1×10^{-3}
**8iv**	3.20[a]	Single peak + trace impurity	390.1755	0.0
**8v** (known)	-	-	350.1070	-0.5×10^{-3}
**8vi**	6.68[b]	Single peak	348.0922	-2.2×10^{-3}
**8vii** (known)	-	-	368.0973	-2.5×10^{-3}
**8viii**	3.06[a]	Single peak + trace impurity	402.0583	-1.0×10^{-3}
**8ix**	3.12[a]	Single peak	436.0193	0.1×10^{-3}
**8x**	3.02[a]	Single peak	382.1129	0.5×10^{-3}
**8xi**	2.98[a]	Single peak + trace impurity	398.1078	0.4×10^{-3}
**8xii**	4.28[b]	Single peak + trace impurity	411.1395	1.0×10^{-3}
**8xiii**	4.56[b]	Single peak + trace impurity	383.1082	0.2×10^{-3}
**8xiv**	5.02[b]	Single peak + trace impurity	320.0609	0.7×10^{-3}
**8xv**	6.22[b]	Single peak + trace impurity	432.0592	-0.2×10^{-3}
**8xvi**	6.73[b]	Single peak	378.1391	0.8×10^{-3}
**8xvii**	6.48[b]	Single peak + trace impurity	468.0361	-0.5×10^{-3}
**8xviii**	6.92[b]	Single peak	344.1781	0.3×10^{-3}
**8xix**	6.82[b]	Single peak + trace impurity	324.2327	0.5×10^{-3}
**8xx**	6.53[b]	Single peak	340.2277	-0.1×10^{-3}
**8xxi**	6.77[b]	Single peak + trace impurity	398.1078	1.2×10^{-3}

[a] Samples were separated using an Agilent 1100 HPLC with photodiode array detector, using a Phenomenex Gemini C18 110A 5 μM 3.0 × 50 mm column and analyzed by electrospray positive mode on a Bruker microTof. A gradient from 95% Water / MeCN to 95% MeCN / Water over 6 min at a flow rate of 0.8 ml min^{-1} was applied.

[b] The samples were run in electrospray positive mode on a Micromass LCT, which is a high performance orthogonal acceleration reflecting TOF mass spectrometer, coupled to a Waters 2795 HPLC and a Waters 2996 photodiode array detector, using an XTerra® RP 18 5 μM, 3.0 × 50 mm column. A Capillary Cone voltage of 3500V, an Extraction Cone voltage of 30V and Rf lens of 300V was used. A gradient from 98% (Water / 0.0%5 NH_4OH) / MeCN to 98% (MeCN / 0.0%5 NH_4OH) / Water over 7 minutes at a flow rate of 1 ml min^{-1} was applied.

References

[S1] A. Stroba, *J. Med. Chem.,* **2009**, *52*, 4683-4693.

[S2] X.-H. Gu, H. Yu, A. E. Jacobson, R. B. Rothman, C. M. Dersch, C. George, J. L. Flippen-Anderson, K. C. Rice, *J. Med. Chem.,* **2000**, *43*, 4868-4876.

[S3] X.-L. Wu, G.-W. Wang, *Tetrahedron,* **2009**, *65*, 8802-8807.

[S4] (a) P.-L. Zhao, C.-L. Liu, W. Huang, Y.-Z. Wang, G.-F. Yang, *J. Agric. Food Chem.,* **2007**, *55*, 5697-5700; (b) H.E. Blackwell, M.D. Bowman, J. C. Stringer, US Pat. Appl. Publ. (2008), US 20080009528 A1 20080110.

[S5] (a) C.M. Brennan, I. Hunt, T. C. Jarvis, C. D. Johnson, P. D. McDonnell, *Can. J. Chem.,* **1990**, *68*, 1780-1785; (b) S. Radhika, S.K. Nayak, N. L. Chavan, *Tetrahedron Letts.,* **2006**, *47*, 7323-7326; (c) S. Nayak, *Synth. Commun..,* **2006**, *36*, 1307-1315; (d) L.D. Hicks, A.J. Fry, V. C. Kurzweil, *Electrochim. Acta,* **2004**, *50*, 1039-1047; (e) U. Shadakshari, S. Nayak, *Tetrahedron.,* **2001**, *57*, 8185-8188; (f) J.P. Bradley, T.C. Jarvis, C. D. Johnson, P. D. McDonnell, T. A. Weatherspoon, *Tetrahedron Letts.,* **1983**, *24*, 2851-2854.

[S6] R.T. Lalonde, B.A. Horenstein, K. Schwendler, R.C. Fritz, R.A. Florence, I. Ekiel, I.C.P. Smith, *J. Org. Chem.,* **1983**, *48*, 4049-4052.

[S7] J. Cossy, *Synlett.,* **2003**, *10*, 1515-1517.

[S8] K.P. Boegesoe, *J. Med. Chem.,* **1983**, *26*, 935-947.

[S9] . H. Yu, *J. Med. Chem.,* **2004**, *47*, 2624-2634.

[S10] X.-H. Gu, *J. Med. Chem.,* **2000**, *43*, 4868-4876.

[S11] K. Bogeso, A. V. Christensen, J. Hyttel, T. Liljefors, *J. Med. Chem.,* **1985**, *28*, 1817-1828.

[S12] W.M. Clark, A. Kassick, M.A. Plotkin, A.M. Eldridge, I. Lantos *Org. Lett.,,* **1999**, *1*, 1839-1842.

[S13] L.F. Silva, F.A. Siqueira, E.C. Pedrozo, F.Y.M. Vieira, A.C. Doriguetto *Org. Lett.,,* **2007**, *9*, 1433-1436.

[S14] M. Froimowitz, M.; K-M. Wu, A. Moussa, R. M. Haidar, J. Jurayj, C. George, E. L. Gardner, *J. Med. Chem.,* **2000**, *43*, 4981-4992
